# Role of genomic architecture in the expression dynamics of long noncoding RNAs during differentiation of human neuroblastoma cells

**DOI:** 10.1186/1752-0509-7-S3-S11

**Published:** 2013-10-16

**Authors:** Arsen O Batagov, Aliaksandr A Yarmishyn, Piroon Jenjaroenpun, Jovina Z Tan, Yuichiro Nishida, Igor V Kurochkin

**Affiliations:** 1 Department of Genome and Gene Expression Data Analysis, Bioinformatics Institute, 30 Biopolis Str, Singapore, 138671; 2 Laboratory for Computational and Experimental Systems Biology Group, RIKEN Genomic Sciences Centre, 1-7-22 Suehiro-cho, Tsurumi-ku, Yokohama, Kanagawa 230-0045, Japan

## Abstract

**Background:**

Mammalian genomes are extensively transcribed producing thousands of long non-protein-coding RNAs (lncRNAs). The biological significance and function of the vast majority of lncRNAs remain unclear. Recent studies have implicated several lncRNAs as playing important roles in embryonic development and cancer progression. LncRNAs are characterized with different genomic architectures in relationship with their associated protein-coding genes. Our study aimed at bridging lncRNA architecture with dynamical patterns of their expression using differentiating human neuroblastoma cells model.

**Results:**

LncRNA expression was studied in a 120-hours timecourse of differentiation of human neuroblastoma SH-SY5Y cells into neurons upon treatment with retinoic acid (RA), the compound used for the treatment of neuroblastoma. A custom microarray chip was utilized to interrogate expression levels of 9,267 lncRNAs in the course of differentiation. We categorized lncRNAs into 19 architecture classes according to their position relatively to protein-coding genes. For each architecture class, dynamics of expression of lncRNAs was studied in association with their protein-coding partners. It allowed us to demonstrate positive correlation of lncRNAs with their associated protein-coding genes at bidirectional promoters and for sense-antisense transcript pairs. In contrast, lncRNAs located in the introns and downstream of the protein-coding genes were characterized with negative correlation modes. We further classified the lncRNAs by the temporal patterns of their expression dynamics. We found that intronic and bidirectional promoter architectures are associated with rapid RA-dependent induction or repression of the corresponding lncRNAs, followed by their constant expression. At the same time, lncRNAs expressed downstream of protein-coding genes are characterized by rapid induction, followed by transcriptional repression. Quantitative RT-PCR analysis confirmed the discovered functional modes for several selected lncRNAs associated with proteins involved in cancer and embryonic development.

**Conclusions:**

This is the first report detailing dynamical changes of multiple lncRNAs during RA-induced neuroblastoma differentiation. Integration of genomic and transcriptomic levels of information allowed us to demonstrate specific behavior of lncRNAs organized in different genomic architectures. This study also provides a list of lncRNAs with possible roles in neuroblastoma.

## Background

The transcriptome analysis studies of the past decade revealed that only a small proportion of mammalian genomes (less than 2%) is transcribed into protein coding mRNAs [1,2]. The remaining non-coding part of the genome on the other hand is extensively transcribed into various classes of non-coding RNAs. Among them small regulatory RNAs, such as microRNAs and siRNAs, have been extensively studied. However, the largest fraction of the non-coding transcriptome is represented by long non-coding RNAs (lncRNAs), which are defined as transcripts having size larger than 200 nucleotides [3,4]. This vast class of non-coding RNAs still remains poorly understood and its functionality continues to be a subject of debate. However, evidence is growing that many lncRNAs are important functional molecules involved in various regulatory processes. The functional lncRNAs demonstrate precise spatiotemporal patterns of expression and often exhibit specific cellular localization [5-8]. So far, lncRNAs have been shown to be associated with various biological and pathological processes, such as cell differentiation [9], embryonic development [10], immune response [11], and cancer [12]. Several insights have been gained into molecular mechanisms of lncRNA activity, specifically some lncRNAs have been shown to regulate gene expression by chromatin remodeling [13], modulation of transcription factors [14,15], translation [16], and RNA stability [17]. LncRNA genes are often arranged into complex transcriptional loci with the protein coding genes, from which they are expressed in a coordinated fashion [6,18,19]. Such complex loci may include overlapping architecture, such as cis-antisense, intronic, bidirectional, as well as non-overlapping with genes located in their close vicinity. Some lncRNA genes associated with protein-coding genes on genomic level encode lncRNAs cooperating with proteins on the transcriptome and proteome levels. A number of studies have demonstrated functional relationship between lncRNAs and their associated protein coding genes [15,20-22]. Several recent reports focused on predicting functions of lncRNAs from their co-localization with protein coding genes applying integrated analysis of transcriptome [5,6,19]. The present work extends the previous studies by detailing both characterization of lncRNA genomic architecture types and their relation to dynamics of lncRNA transcripts.

We investigated expression of lncRNAs during differentiation of SH-SY5Y neuroblastoma cell line induced by retinoic acid (RA). Using our custom microarray chip, for the first time we measured the dynamics of lncRNA expression in this model system of neuronal differentiation. The most detailed of existing annotations of lncRNA genomic architecture allowed us to discriminate 19 lncRNA classes and to evaluate expression dynamics for each individual class. We integrated this data with our previous work on dynamics of protein expression measured at the same conditions to identify potential architecture-dependent regulatory mechanisms coupling expression of lncRNAs and neighboring proteins.

## Results

### Classification of lncRNA genes

**Figure 1:**
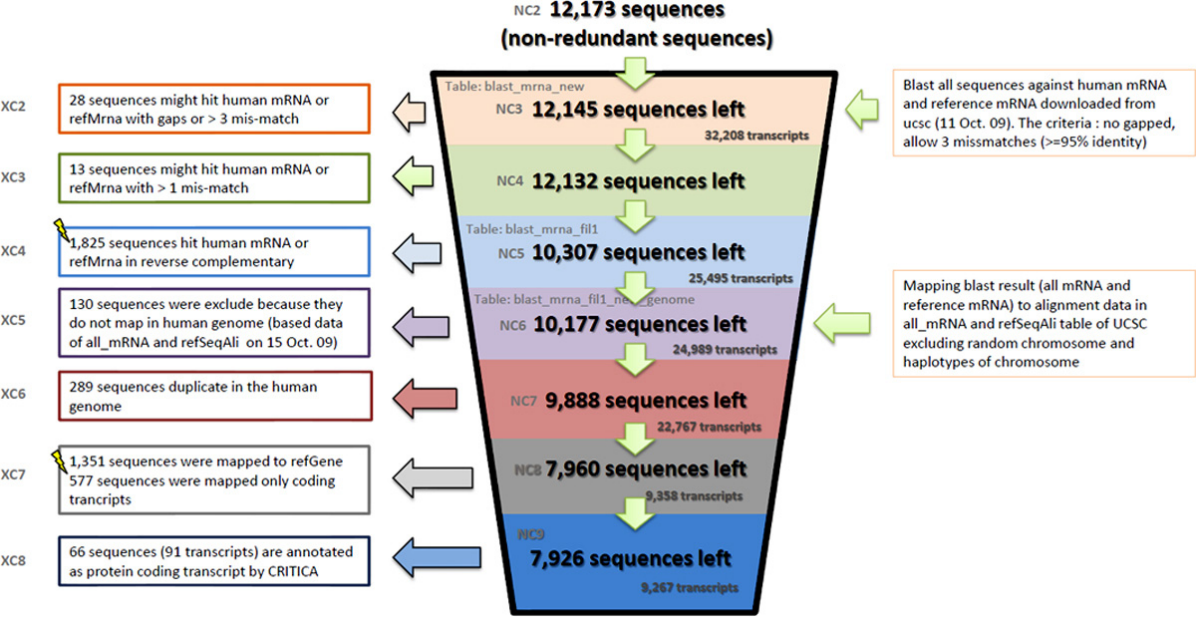
**Computational pipeline for annotation of custom microarray probes targeting lncRNAs**. The results of sequential filtration in steps (top to bottom) are shown in the coloured boxes on the central panel. The summary on the probes filtered out at each filtration step is shown at the left panel. Descriptions of the procedures are presented on the right panel.

LncRNAs were identified among the genes of human genome build 36 annotated in H-Invitational database [23]. Absence of coding regions in the genes was verified using CRITICA software, a hybrid method that combines comparative analysis with statistical analysis of coding sequences [24]. The final list included 9,267 lncRNA genes (Figure 1).

**Figure 2:**
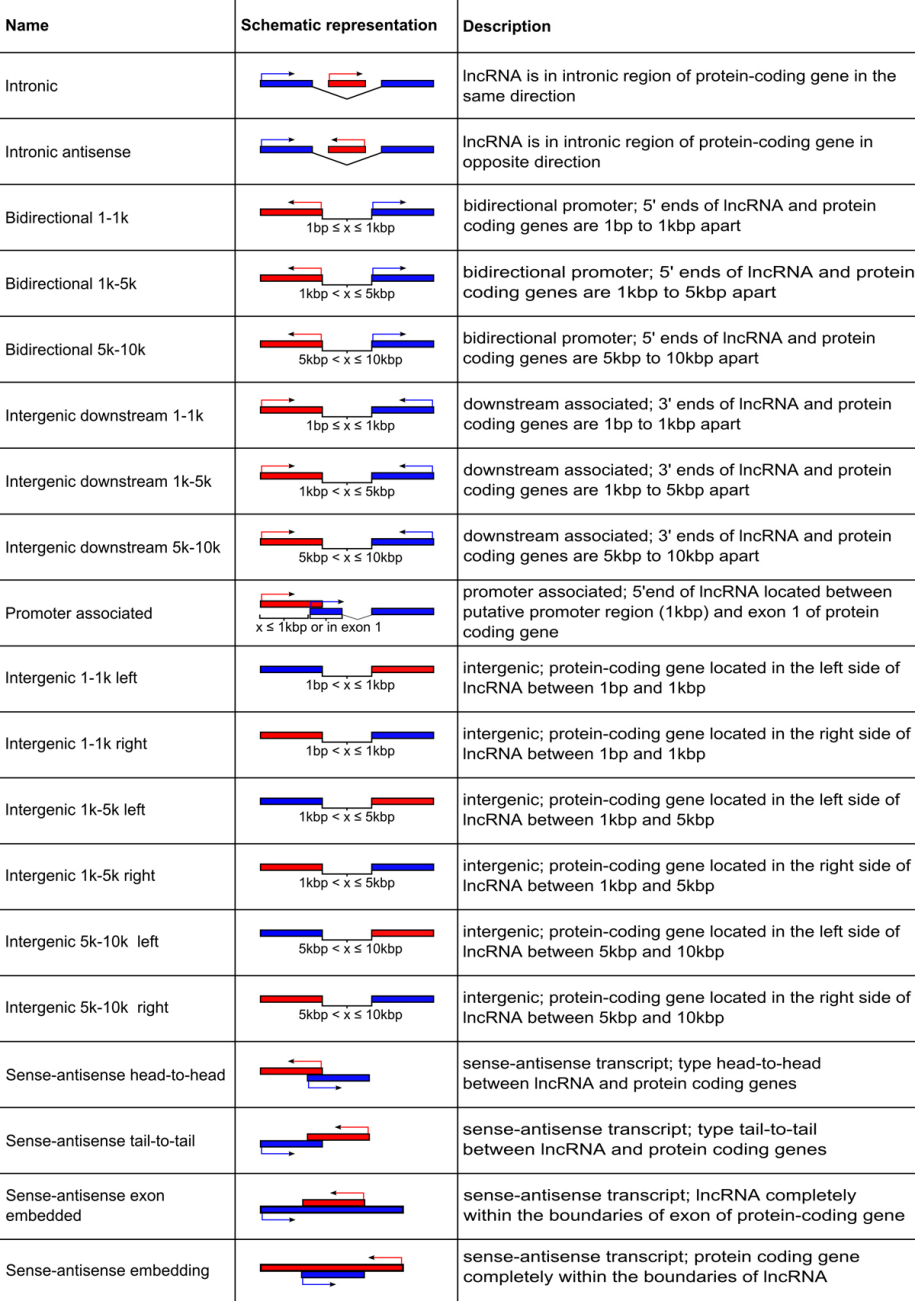
**Classification of genomic architecture classes involving gene pairs of lncRNAs and protein-coding genes**. The 19 architectures (in rows) describing co-localization of lncRNAs with protein-coding genes. Left column contains the name of the architecture; central column contains a schematic drawing representing; right column contains a short description of the architecture. Note: the 'left' and the 'right' architecture classes represent the localization of the protein-coding gene boundaries relative to the lncRNA gene boundaries on the plus DNA strand, regardless of the sense or antisense orientation of both genes.

The genes were further classified by their association with neighbouring protein-coding genes by genomic architecture (GA). The closest protein coding genes in sense or antisense orientation within 10 kbp vicinity of the lncRNA genes were identified as associated with particular lncRNAs. The associated lncRNA-protein coding gene pairs were further classified by their GA into five major groups: antisense, intergenic, promoter-associated and intronic, relative to the protein-coding genes. Each group was sub-classified, yielding in total 19 classes of lncRNA-protein gene association types (Figure 2). Antisense pairs were sub-classified into embedding, exonic, head-to-head and tail-to-tail classes. Intergenic pairs were sub-classified into upstream-associated and downstream-associated. Each of these two classes was further classified by the respective intergenic distance into three subclasses: 1 kbp, 5 kbp and 10 kbp. Being of a of particular interest, the pairs sharing bidirectional promoters were similarly sub-classified into 1, 5 and 10 kbp - distant. The exonic pairs were sub-classified into purely exonic and embedding. The latter class included cases when lncRNA genes were located within the genomic boundaries of the associated proteins and, at the same time, were overlapping with both exonic and intronic sequences. Embedding, exonic and intronic pairs were sub-classified into sense and antisense subtypes, relative to the protein-coding gene.

**Figure 3:**
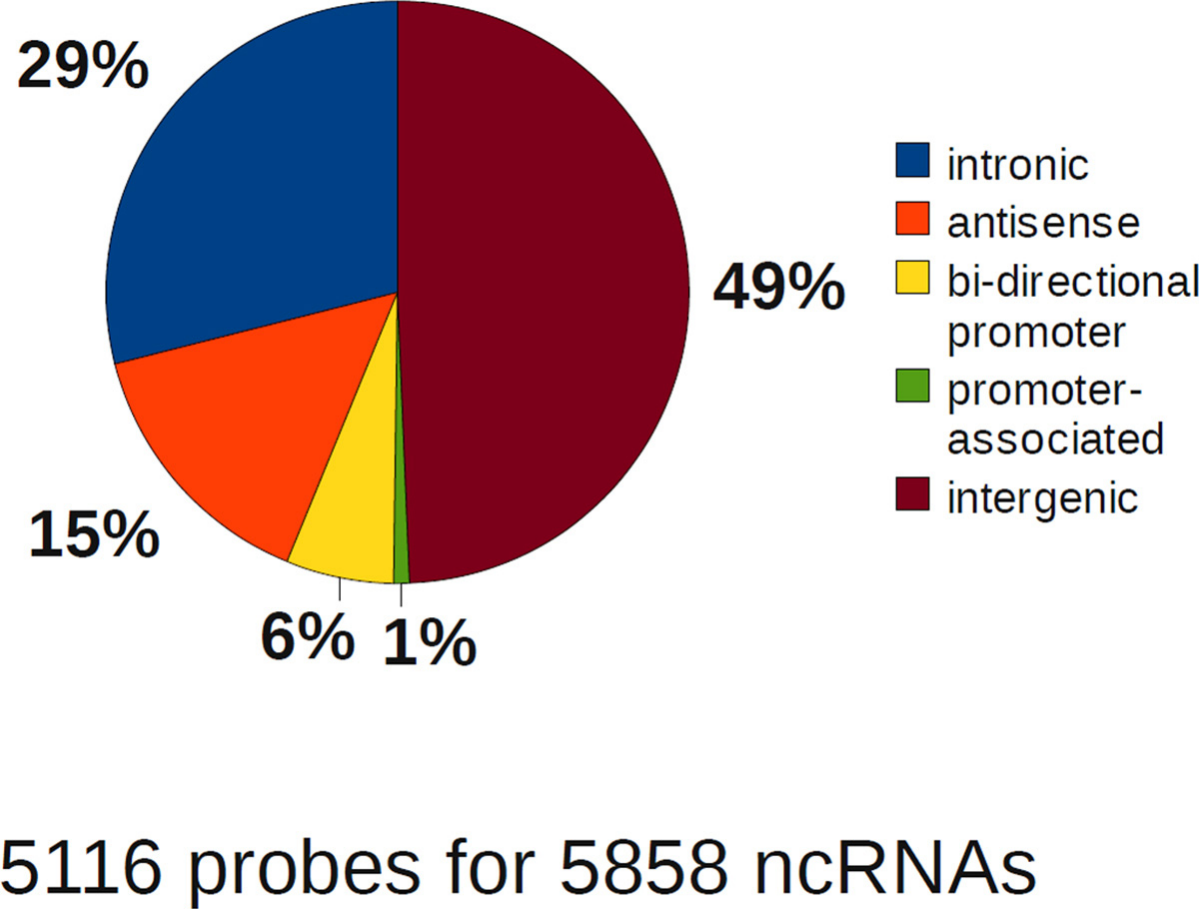
**Frequency distribution of the genomic architecture super-classes of the lncRNAs measured by the custom microarray chip**. The 19 distinct genomic architecture classes (see Figure 2) were generalized into 5 super-classes (intronic antisense bidirectional promoter, intronic and intergenic orientations) by lncRNA orientation, representing the overall topology of lncRNA-protein coding genes co-localization. Frequencies of all lncRNAs measured in the present study are given.

In total, 5,116 lncRNA genes were found to be associated with protein coding genes, according to the above criteria. Among them the fractions of intergenic, intronic, antisense and promoter-associated lncRNA genes were 49%, 29%, 15% and 7%, respectively (Figure 3).

Surprisingly, gene ontology (GO) analysis revealed evidence that the architecture of lncRNA-protein coding gene pairs may be related to functional specialization of the proteins in these pairs. The list of significantly enriched GOs specific to certain architecture types included genes associated with cell differentiation, embryogenesis, signalling pathways, and cytoskeleton. For more details, see Section 1.1 of the Additional file 1.

### Differential expression of lncRNAs and associated protein coding genes

**Figure 4:**
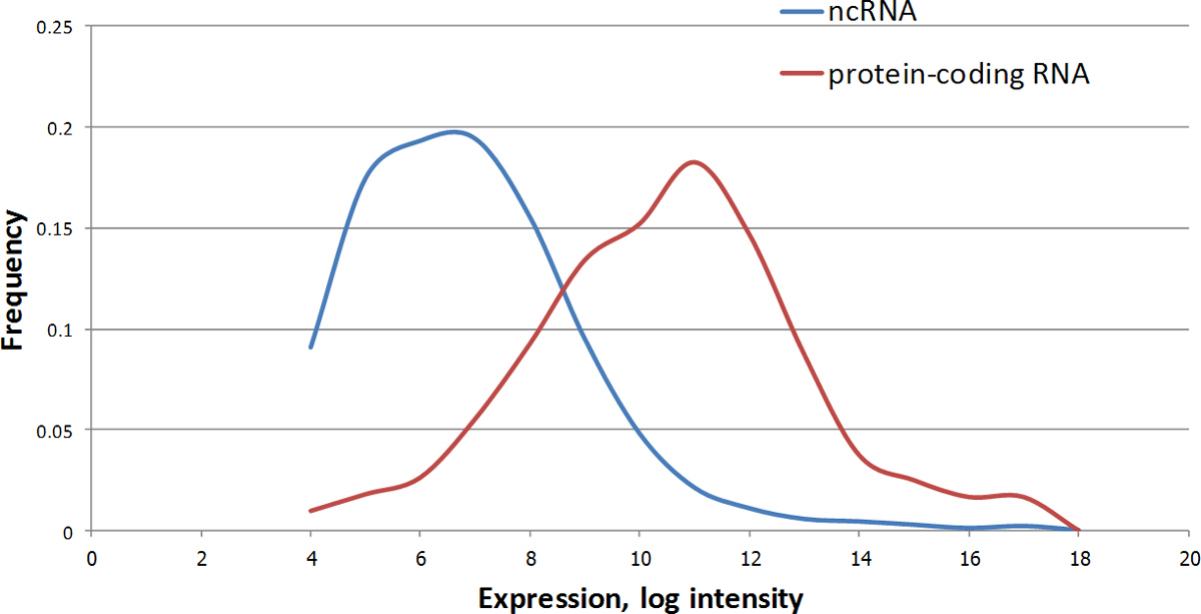
**Distribution of expression values of lncRNAs and protein-coding genes**. Smoothed densities of lncRNA (blue line) and protein-coding genes (red line) mean expression values measured in the course of RA-induced neuroblastoma cells differentiation in the present study. The data have been derived from the analysis of the custom microarray described in this study. In addition to 11,564 probes specific to lncRNAs, the array also included 728 probes matching 446 protein-coding genes.

To measure the expression of protein coding gene-associated lncRNAs during neuroblastoma differentiation, a custom microarray chip was designed and implemented using Agilent platform. Two biological replicates of differentiating neuroblastoma cells were screened at four time points (0, 6, 24 and 120 hours) after a single-time stimulation by RA. Confirming previous studies, the overall expression values of lncRNAs were observed to be lower than the values of mRNAs (Figure 4).

**Figure 5:**
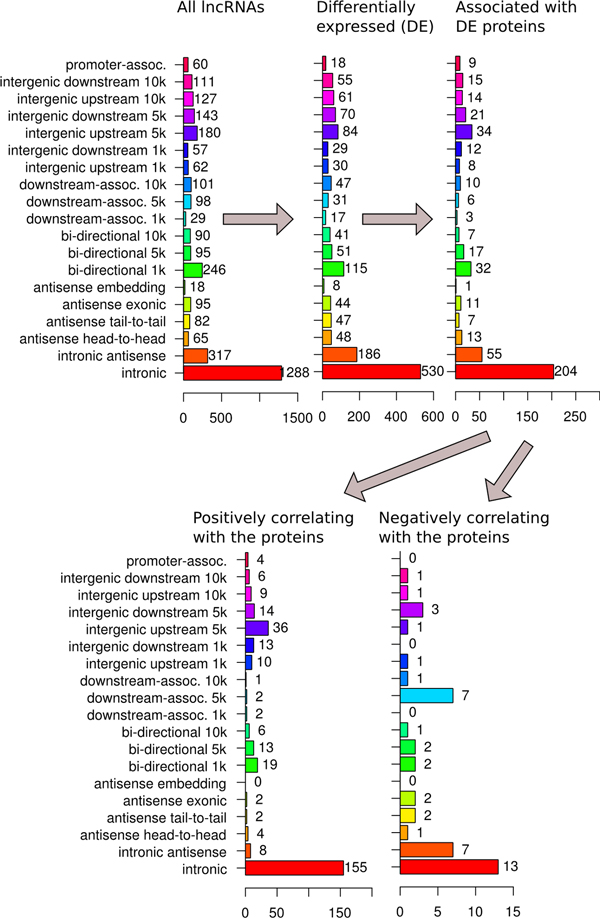
**Protein-associated differentially expressed lncRNAs classified by their genomic architecture**. Distribution of protein-associated lncRNAs by their genomic architecture classes (see Figure 2) in a sequence of filtration steps (shown with arrows) yielding the lists of differentially expressed (fold change cutoff 1.5) positively and negatively correlating (Kendall's τ≥0.6) lncRNA-protein pairs. The information for the protein coding genes has been derived from Affymetrix U133-Plus2.0 microarrays reported in [26].

The distribution of all differentially expressed lncRNAs revealed an increase in the fraction of transcripts with antisense GA from 18% to 22% (Figure 5). Significant increase was observed for lncRNAs with antisense head-to-head GA relative to that with intronic (*P *= 1.61·10^-6^, fold enrichment FE = 1.8), 1 kbp-distant bidirectional promoter (*P *= 0.025, FE = 1.6), 5 kbp-distant downstream-associated (*P *= 3.71·10^-5^, FE = 2.3), 5 kbp-distant intergenic downstream (*P *= 0.042, FE = 1.6), and promoter-associated (*P *= 2.91·10^-4^, FE = 2.5) GAs (the calculation procedure is detailed in Satistical analysis section of Methods). Intronic antisense lncRNA were over-represented in comparison with intronic (*P *= 2.51⋅10−6, FE = 1.4) and promoter-associated (*P *= 0.013, FE = 2.0) lncRNAs. These observations are consistent with the well-known fact that pairs of complementary transcripts may regulate the stability of their counterparts [25].

Next, we tested whether the influence of lncRNA GA is specific to differential expression of lncRNAs or whether it might be related with the expression of the associated protein coding genes. Therefore, differentially expressed lncRNAs associated with differentially expressed protein coding genes were compared with all the differentially expressed lncRNAs, as well as with those that correlate insignificantly with the associated protein coding genes. To identify possible functional connections between the lncRNAs and their associated protein coding genes, in the cases when the expression of the lncRNA-protein pairs correlate over time, GA frequencies were evaluated separately.

Contrary to the general tendency of differentially expressed lncRNAs, the fraction of antisense GAs in positively correlating lncRNA-protein coding gene pairs decreased from 12% to 5% (Figure 5). The ratios between the individual antisense GA frequencies in all differentially expressed lncRNAs and those lncRNAs that positively correlatewith expression of protein coding genes were 4.4 for intronic, 3.5 for exonic, 2.2 for tail-to-tail and 2.1 for head-to-head architectures. In comparison with intronic architecture, the differences were strongly significant (P-values ranging from 6.15⋅10^−9 ^to approximately zero with FE from 6.5 to 12.2). In contrast, among negatively correlating lncRNA-protein coding genes pairs the frequency of intronic, exonic, tail-to-tail and head-to-head antisense GAs was 6, 6.8, 6.8 and 1.7-times higher. Except for the head-to-head GA, the fraction of the antisense architecture types was higher in negatively correlating lncRNAs in comparison with all differentially expressed lncRNA-protein coding pairs. The results suggest that antisense mode of architecture-dependent regulation for lncRNAs is largely negative. GO analysis of the protein-coding genes postitively correlating with their paired lncRNA genes did not reveal significant associations with biologcal functions specific to any given architecture.

To find whether the observed correlations between the expression levels of lncRNAs and protein coding genes are specific to certain temporal expression patterns, we analyzed their dynamics in the course of RA-induced cell differentiation.

### Dynamical modes of lncRNA expression

**Figure 6:**
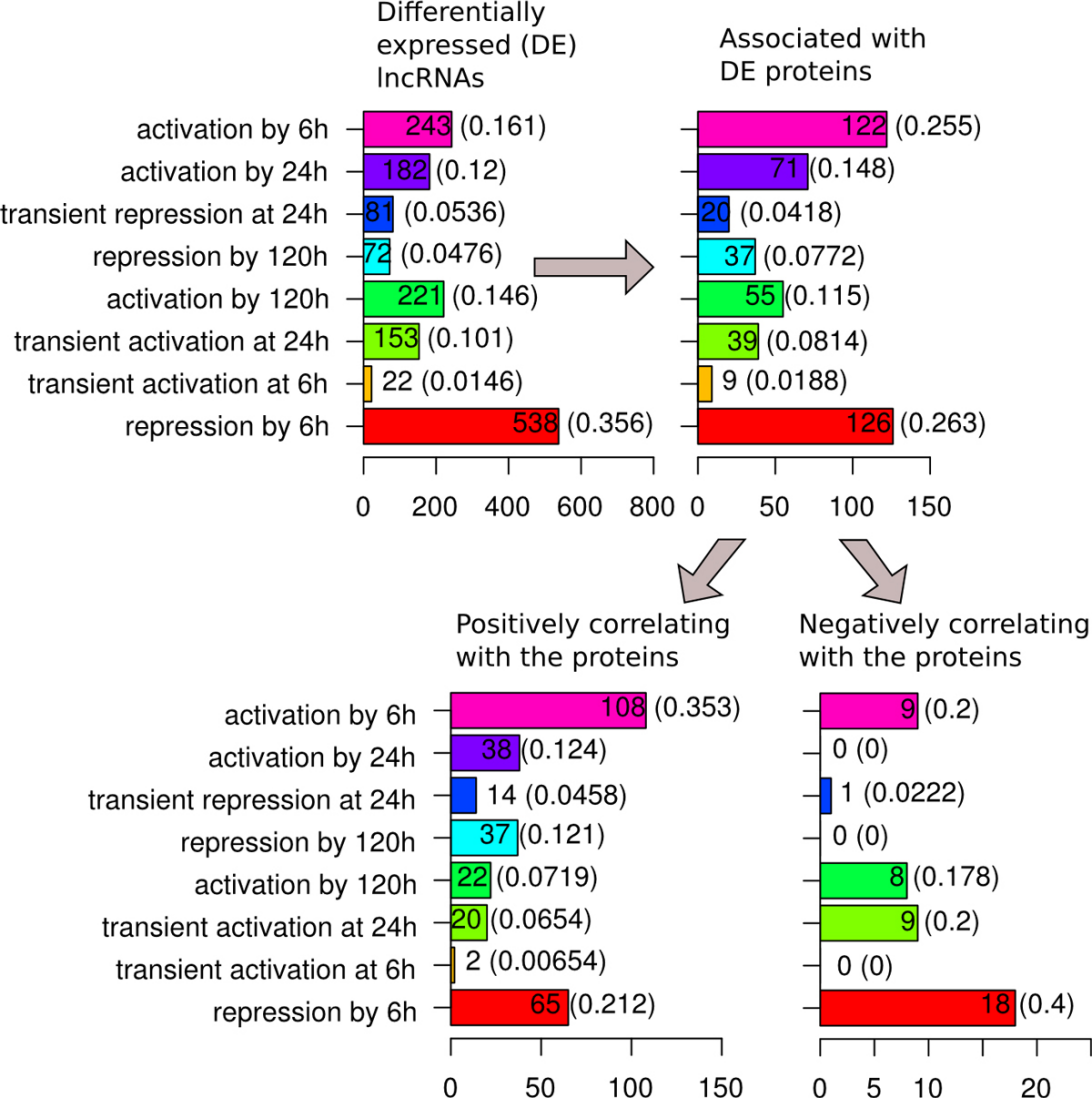
**Protein-associated differentially expressed lncRNAs classified by their "expression rate" dynamic modes**. Distribution of protein-associated lncRNAs by their "expression rate" dynamic modes (see Additional File 2) in a sequence of filtration steps presented on Figure 5.

Expression of lncRNAs and protein coding genes was analyzed at four time points in the course of RA-induced neuroblastoma cells differentiation. Dynamical patterns (modes) of lncRNA expression were discriminated: i) by the time point when a given lncRNA activation/repression is observed (Additional file 2, panel A) and ii) by the time interval of increased/decreased expression of a given lncRNA (Additional file 2, panel B). These two types of modes were named as "rate" and "magnitude", respectively. Analysis of the "rate" modes revealed that most of the studied lncRNAs were either activated or repressed already by hour six of cell differentiation and followed such trend until the end of the experiment (Figure 6). These two modes together comprised 52% of the differentially expressed lncRNAs.

The largest increase in the frequency of mode occurrence was observed for the lncRNAs repressed by 120 h, followed by the ones activated by 6 h. Among the differentially expressed lncRNAs associated with the differentially expressed protein coding genes the frequencies of these modes were 1.6-times higher than those among the all differentially expressed lncRNAs. LncRNAs transiently activated at 6h and permanently activated by 24 h were the other two classes whose frequency was higher among the protein-associated differentially expressed lncRNAs, 1.3- and 1.2-times, respectively. The largest frequency decrease (1.3- times) was observed among lncRNAs permanently repressed by 6 h, followed by the ones activated by 120 h. The above tendencies were more clear when the protein coding gene-associated differentially expressed lncRNAs had been classified into positively and negatively correlating. For positively-correlating lncRNA genes the mode with the largest fraction increase (1.6-times) was repression by 120 h, followed by the mode of permanent activation by 6 h. At the same time, the fraction of lncRNAs activated by 120 h, as well as permanently repressed by 6 h further decreased, 1.6- and 1.2-times, respectively.

Surprisingly, the modes with the highest increase among the positively-correlating lncRNAs were strongly under-represented among the negatively correlating lncRNAs and vice-versa. As such, for the negatively correlating lncRNAs, the fraction of lncRNAs activated by 120 h increased 1.6-times, and those permanently repressed by 6 h increased 1.5-times. On the contrary, the fraction of lncRNAs permanently activated by 6 h decreased 1.3-times, while lncRNAs activated by 24 h were completely absent. Also, the frequency of lncRNAs transiently activated at 24 h increased 2.5-times.

**Figure 7:**
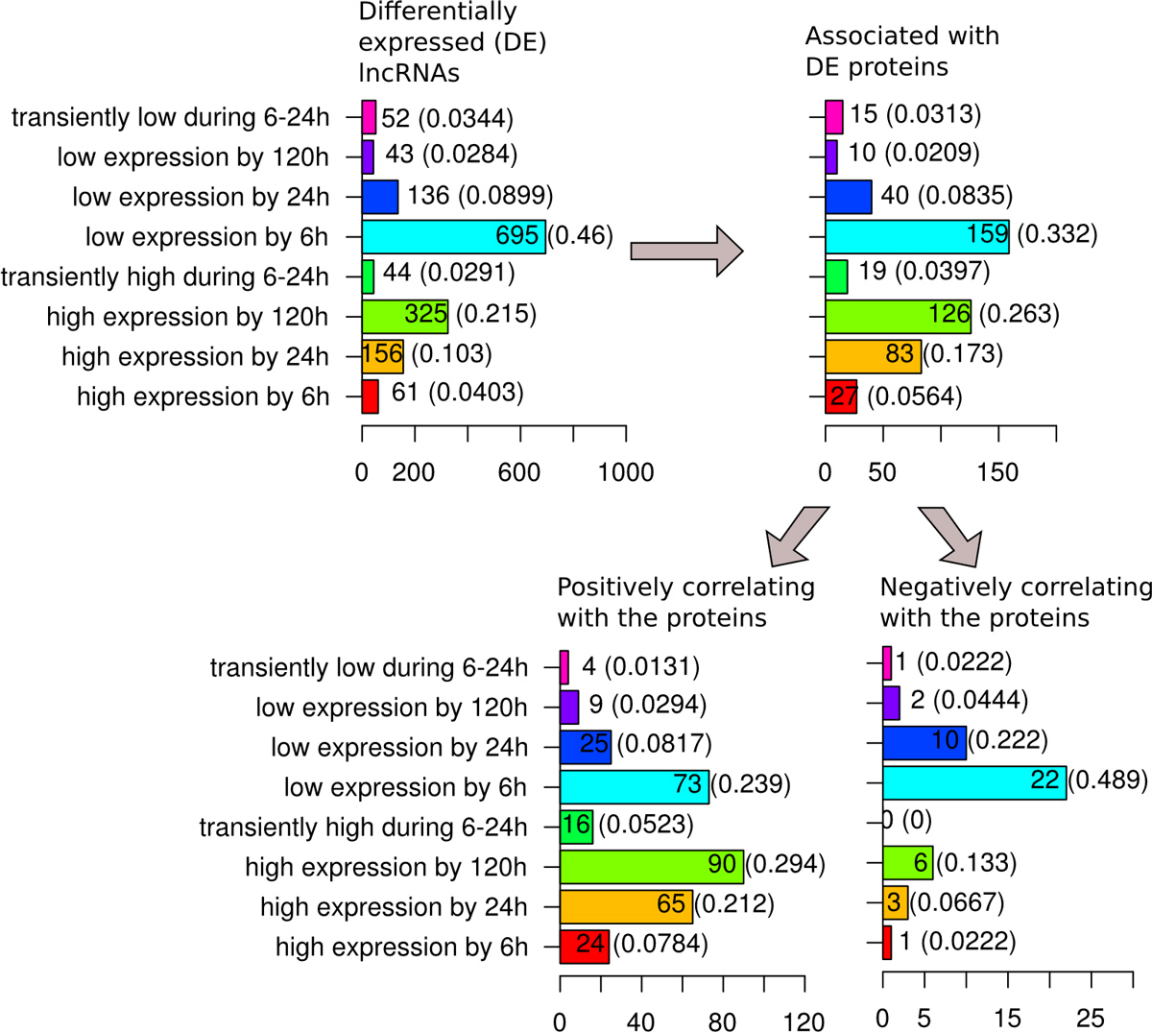
**Protein-associated differentially expressed lncRNAs classified by their "expression magnitude" dynamic modes**. Distribution of protein-associated lncRNAs by their "expression magnitude" dynamic modes (see Additional file 2) in a sequence of filtration steps presented on Figure 5.

Analysis of the "magnitude" modes confirmed and further clarified the dynamic patterns described above (Figure 7). Among all differentially expressed lncRNAs the most frequent modes were "decreased expression by 6 h" (46%) and "increased expression by 120 h" (22%). Overall, protein-associated differentially expressed lncRNAs revealed an increase in the increased-expression modes: upregulation by 24 h (1.7-times increase in fraction), upregulation by 6 h (1.4-times increase), transiently upregulated during 6-24 h (1.4-times increase), upregulation by 120 h (1.2-times increase). On the contrary, a reduction in the fraction of decreased expression modes was observed. The most notable was a 1.4-times decrease for the lncRNAs with down regulation by 6 h and by 120 h. Except for the lncRNAs with decreased expression by 120 h, the distribution of "magnitude" modes in general reflected the distribution of the "rate" modes.

Similarly, when the differentially expressed lncRNAs positively correlating with their associated protein coding genes were compared with those of all the protein coding gene-associated differentially expressed lncRNAs, a further rise in the fraction of the increased-expression modes was observed. Namely, for the fractions of lncRNAs with increased expression by 6 h, 24 h and 120 h the increase was 1.4-, 1.2- and 1.1-times, respectively. The fraction of lncRNAs with transiently increased expression between 6 h and 24 h increased 1.3-times. At the same time, for most of the downregulated expression modes a decrease in their fraction among the positively correlating lncRNAs was observed. The only exception were lncRNAs with decreased expression by 120 h. Their fraction increased 1.4-times. For lncRNAs positively correlating with their associated protein coding genes, compared to all differentially expressed lncRNAs, the frequency of transcripts activated by 6 h increased 2.2-times, while the frequency of transcripts activated by 120 h and 24 h decreased 2.0- and 1.6-times, respectively. The opposite was observed for the repressed lncRNAs. The fraction of lncRNAs repressed by 6 h decreased 1.7-times, while the fraction of lncRNAs repressed by 120 h increased 2.5-times. See Additional files 3, 4, 5, 6 for more details.

Since the listed differences were strongly statistically significant, the results suggest that GA is linked with lncRNAs positive correlation with the protein coding gene counterparts if the former are induced, or negative correlation if the former are repressed. We also observed that early induced (6 h) lncRNAs tend to be positively correlating with the associated protein coding genes, linked with their induction, while late induction of the lncRNAs more often occurs for lncRNAs negatively correlating with the associated protein coding genes, linked with their repression.

**Figure 8:**
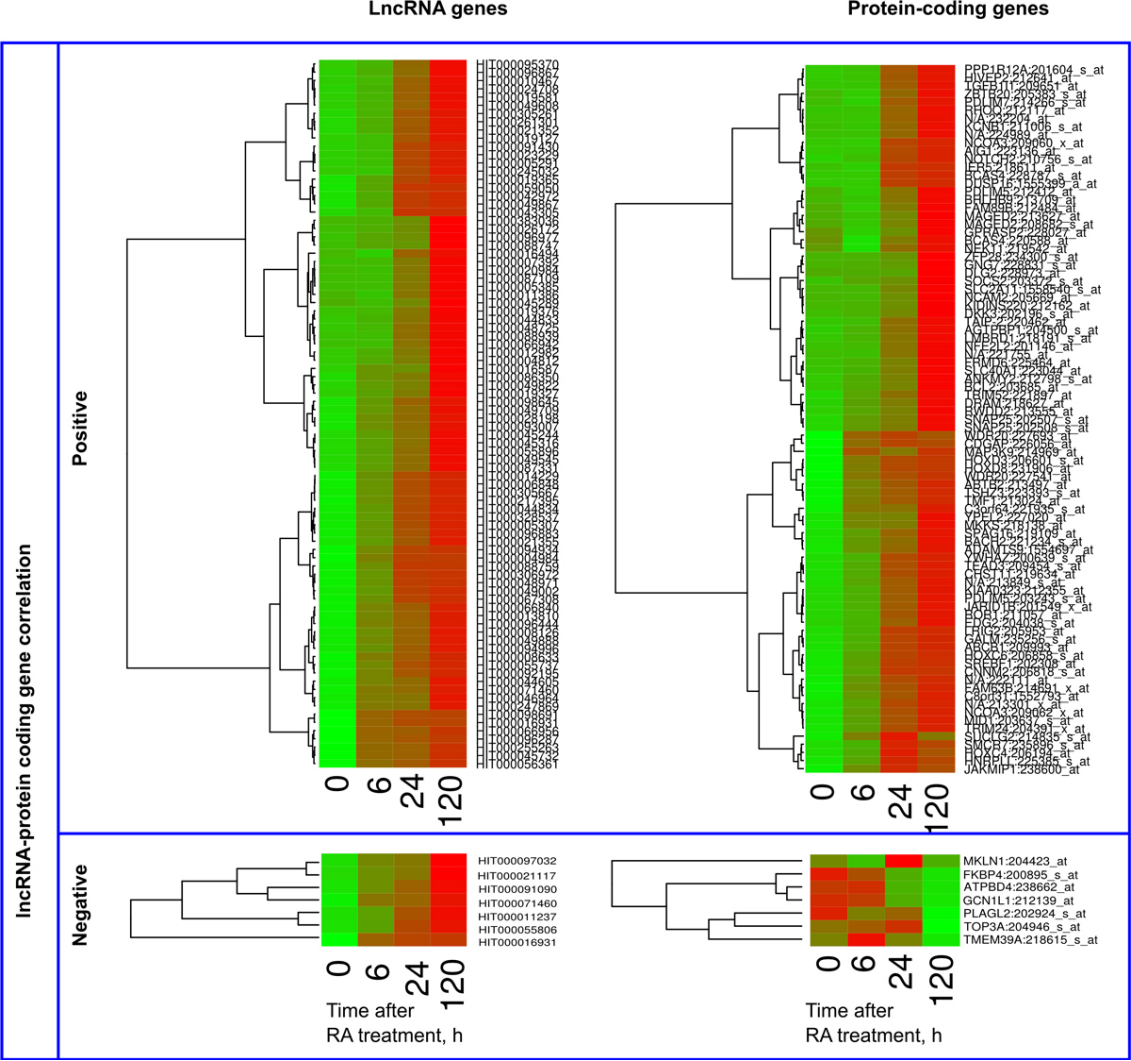
**Cluster diagrams of lncRNA genes activated by 6h and their associated protein coding genes**. Only significantly correlating LncRNA - protein gene pairs are presented. Expression values z-score of individual genes was used to generate the heatmaps. LncRNA and protein coding genes are ordered independently, according to their clustering.

**Figure 9:**
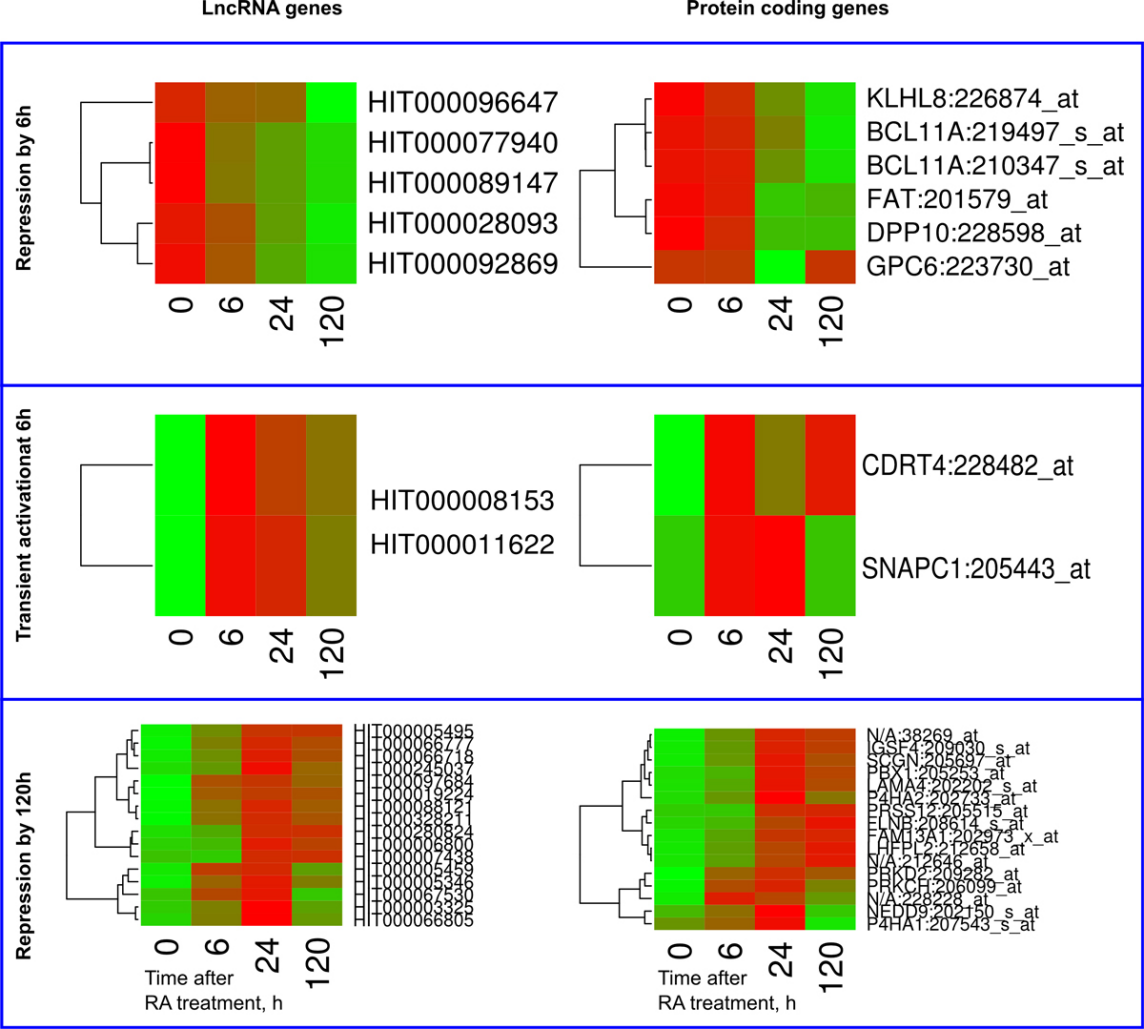
**Cluster diagrams of the lncRNA gene expression revealing different rate repression modes, along with their associated proteins**. Only significantly correlating LncRNA - protein gene pairs are presented. Expression values z-score of individual genes was used to generate the heatmaps. LncRNA and protein coding genes are ordered independently, according to their clustering.

GO analysis revealed that the positively correlating gene pairs activated by 6 h were significantly enriched in muscle development genes (*P *= 6.96·10^-3^), represented by the HOX (HOXC4, HOXC6, HOXD3, and HOXD8) and PDLIM (PDLIM5, PDLIM7) family members, phosphoprotein phoshpatases (DUSP16, NCAM2), and a transcription repressor TMF1 (Figure 8). Three of these genes (TMF1, PDLIM5, PDLIM7) were also associated with actin cytoskeleton. This GO category was significantly enriched among the 6 h-activated positively correlating gene pairs (*P *= 3.61·10^-2^). Two annotated protein-coding genes associated with negatively correlating lncRNAs transiently repressed at 24 h, were PURA and POLR2F. It resulted in a significant gene enrichment for general transcription from RNA polymerase II promoter GO (*P *= 7.39·10^-3^) for this group of genes. LncRNA genes exhibiting repressive expression rate modes did not reveal any significant GOs (See Figure 9 for their heatmaps, along with the ones of the associated positively correlating proteins).

When differential expression was considered regardless the significance of correlation, significant GOs were obsereved for a few more expression patterns. The results below describe the ontologies of protein-coding genes associated with a given type of dynamics of their lncRNA coding neighbors. Analysis of expression rate and magnitude modes demonstrated several waves of functionally specialized lncRNA gene expression. The first wave (0-6 h after RA treatment) permanently activated housekeeping (metabolism, DNA repair, splicing, translation) and non-HOX transcription factor genes. The second wave (24 h) transiently activated genes specific to actin cytoskeleton, nervous system development (including HOX transcription factors), and PDGF pathway. The third wave, visible at 120 h time point, activated genes involved in cell-cell signalling, cation transport, sensory perception, Beta3 adrenergic receptors pathway, Slit/Robo-mediated axon guidance pathway, EGF pathway, integrin signalling, adrelalin biosynthesis and angiogenesis (see details on the GO analysis of dymanic patterns in Section 1.2 of Additional file 1).

All the above associations between gene co-localization and the dynamics of transcription do not have a simple interpretation. Therefore we further investigated the distribution of dynamic modes for each individual GA class. LncRNAs significantly correlating with their associated protein coding genes were compared against the non-significantly correlating ones.

### Association between GAs and expression dynamics

**Figure 10:**
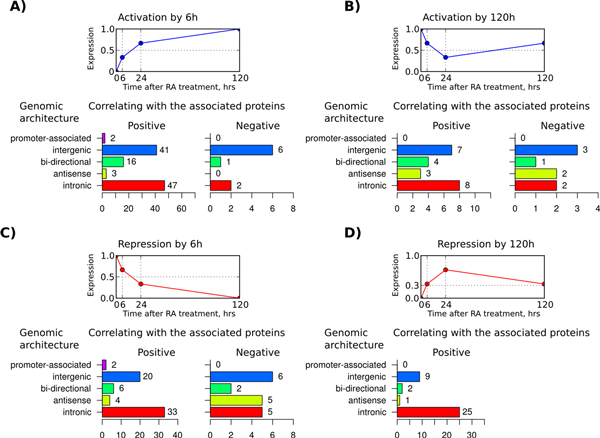
**Distribution of genomic architecture super-classes among lncRNAs of selected dynamic modes**. For lncRNAs, which expression dynamic modes revealed the strongest association with expression of their co-localized protein counterparts, the distribution of genomic architecture super-classes is shown. Four dynamic modes are shown on panels A-D. Each panel includes a schematic representation of the temporal pattern of the dynamic mode (top) and their genomic architecture super-class distribution for lncRNAs positively (bottom-left) and negatively (bottom-right) correlating with their associated proteins, all differentially expressed during RA-induced differentiation of neuroblastoma cells.

In the view of the above observations, modes of rapid (6 h) and delayed (120 h) up- and down-regulation of lncRNAs were of a particular interest. Among the lncRNAs activated by 6 h, intergenic transcripts represented two thirds of negatively correlating lncRNAs (Figure 10A). For the positively correlating lncRNAs this GA class, although abundant, was less frequent than intronic GA (38% vs. 43%). At the same time, among the lncRNAs repressed by 6 h the fractions of intergenic and intronic transcripts were not equal for both positively and negatively correlating lncRNAs (Figure 10C). A different picture was observed for lncRNAs activated and repressed by 120 h. Only positively correlating repressed lncRNAs were characterized with intronic transcripts as the dominant GA class. It represented 68% of positively correlating repressed lncRNAs versus 36% among the induced lncRNAs (Figure 10B and 10D).

**Figure 11:**
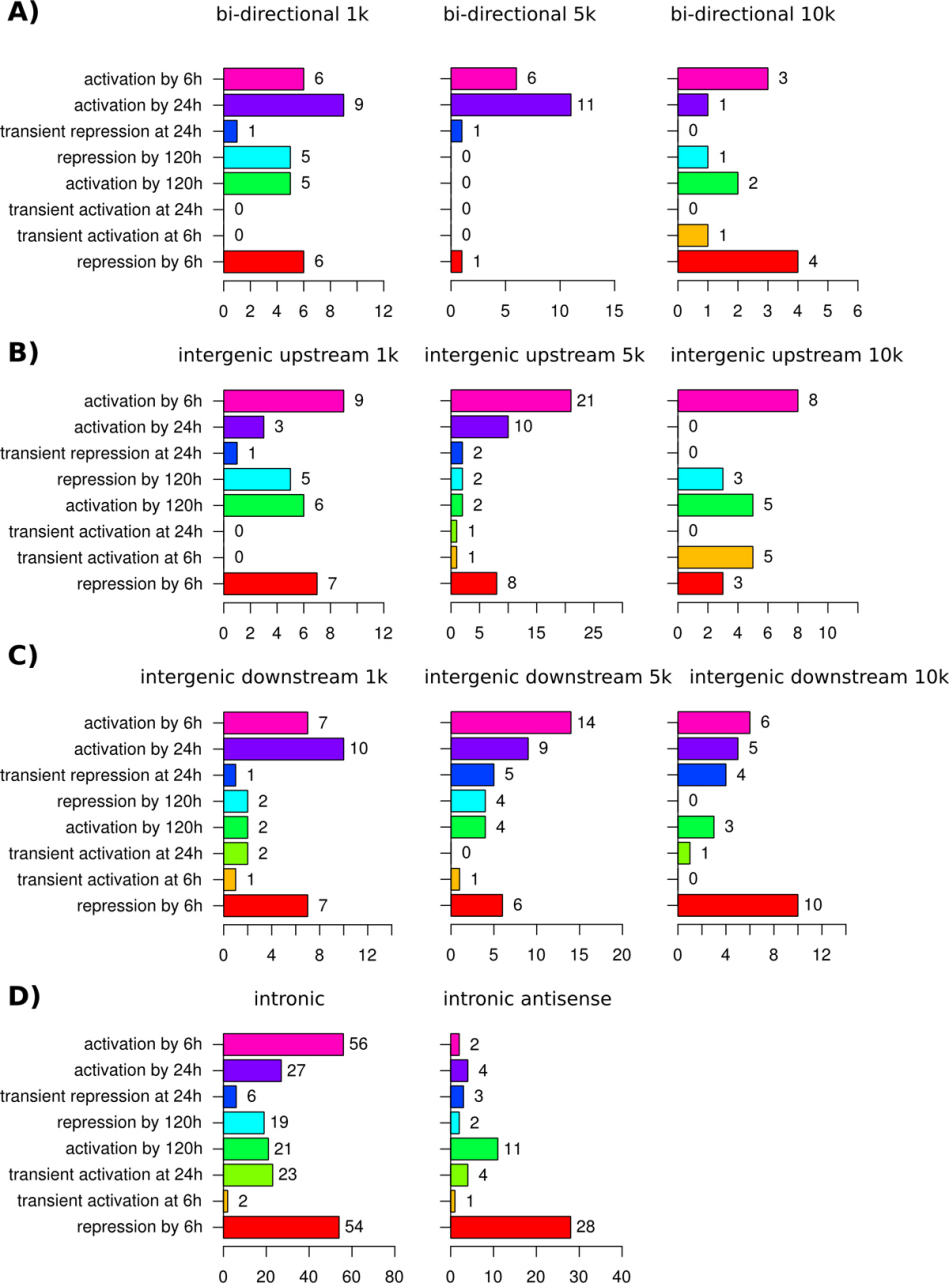
**Distribution of dynamic modes among lncRNAs of selected genomic architecture classes**. Genomic architecture classes revealing associations with specific "expression rate" dynamic modes are presented. Panels A-D combine dynamic mode distributions of lncRNAs belonging to A) bidirectional promoter, B) intergenic upstream, C) intergenic downstream and D) intronic architecture classes.

Remarkably, lncRNAs co-localized with associated proteins in 5 kbp-distant bidirectional promoters, as well as in upstream and downstream intergenic regions, were significantly associated with rapid induction, but not rapid repression (Figure 11A). Most of them positively correlated with the associated protein coding genes (Figure 10A). However, the effect of such induction was rather long term (Additional file 6). At the same time, for lncRNAs localized within 10 kbp from the protein coding genes in bidirectional promoters and downstream intergenic regions, rapid repression was a more common mode. Although the distribution of dynamic modes in correlating protein-associated lncRNAs at 5 kbp and 10 kbp distances in bidirectional promoters and upstream regions was biased towards rapid activation, the majority of rapidly activated and repressed proximal (1 kbp-distant) lncRNAs were equally represented in these GA classes (Figures 11A and 11B). For intergenic downstream lncRNAs a similar tendency was observed. Moreover, for the 1 kbp-distant lncRNAs of this GA class the bias was shifted towards rapid repression with ratio 9:4 (Figure 11C). Notably, among lncRNAs encoded within 5 kbp distance from bidirectional promoters, the bias towards increased expression by 24 h and, especially, 120 h was evident, suggesting existence of a mechanism for the rapid and prolonged activation in the cells.

A clear difference was also observed between intronic sense and intronic antisense lncRNAs. While for the former rapid activation and induction were equally represented, the latter revealed a 14:1 bias towards rapid repression (Figure 11D). For intronic antisense GA the number of lncRNAs activated by 120 h was 5.5-times higher than this number forthe repressedgenes. Among the lncRNAs positively correlating with the associated protein coding genes an opposite bias for delayed regulation was observed. The number of lncRNAs repressed by 120 h was 3.1-times higher than that activated by 120 h (Figures 10B and 10D), although the number of up- and downregulated lncRNAs by this time point was comparable and predominant relatively to the rapid activation modes.

Provided that the majority of intronic antisense lncRNAs had decreased expression level already by 6 h, the results are likely to reflect a slow activation mechanism of lncRNAs not correlating with their associated protein coding genes. At the same time, slowly repressed, rapidly activated and rapidly repressed intronic lncRNAs were mainly positively correlating with their associated protein coding genes. See Additional files 7, 8, 9, 10 for more details.

### Validation of microarray data using qRT-PCR

**Figure 12:**
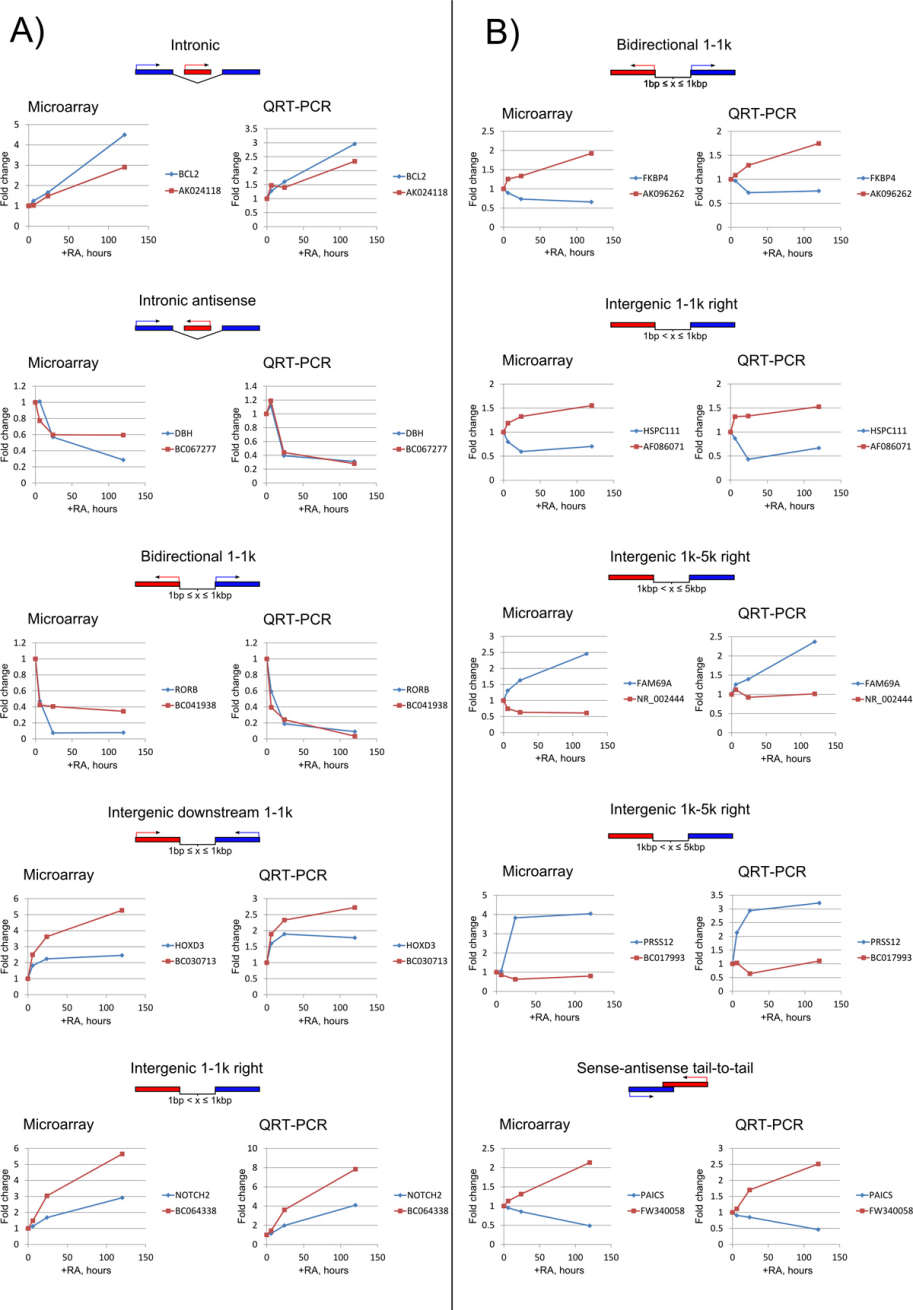
**Validation of selected lncRNA-protein coding gene pairs expression in the course of RA-induced differentiation of SH-SY5Y cells**. For the validation experiments, five positively and five negatively correlating gene pairs as determined by microarrays have been selected. They represented the following major genomic architecture classes: intronic sense, intronic antisense, intergenic, antisense and bidirectional as indicated.

Quantitative RT-PCR (qRT-PCR) was utilized to validate the correlation patterns of lncRNA-protein coding gene pairs obtained with microarrays. For the validation experiments, we selected five positively and five negatively correlating gene pairs. They represented the following major GA classes: intronic sense, intronic antisense, intergenic, antisense and bidirectional. QRT-PCR data for all the gene pairs were concordant with the corresponding microarray data (Figure 12).

## Discussion

In previous studies focusing on the analysis of dynamics of lncRNA expression only four or less architecture classes have been considered. The first study describing genome-wide dynamics of lncRNA expression was published by Ravasi and colleagues in 2006 [27]. Activation of mouse macrophages with LPS led to upregulation of 53 and downregulation of 17 lncRNAs. Several of these transcripts were encoded on the opposite strand of protein-coding genes. Time-course analysis of the activation response revealed no consistent pattern in the expression dynamics of these sense-antisense gene pairs [27]. In a later work, the idea of coordinated regulation of co-localized lncRNA-protein coding gene pairs was tested in a study involving 11 time points of a 16-day course of differentiation of embryoid bodies [6]. The lncRNA-protein coding gene pairs were classified into three categories by their genomic architecture: *cis*-antisense, bidirectional promoter-associated and intronic. Using Pearson's correlation analysis, the study demonstrated that bidirectional and intronic-associated, but not *cis*-antisense classes of transcript pairs tend to correlate positively. Interestingly, their randomized controls demonstrated a strong bias towards positive correlation coefficients in *cis*-antisense pairs and negative correlation for intronic and bidirectional architectures. At the same time, existence of both positively and negatively correlating examples of lncRNA-protein coding gene pairs was noted.

Our study confirms the previous observations in that intronic and bidirectional gene pairs tend to correlate positively. A detailed analysis of genomic architecture allowed us for the first time to demonstrate that lncRNAs in downstream-associated orientations towards protein coding genes tend to correlate negatively with the protein coding genes, while intronic antisense transcripts were equally represented as positively and negatively correlating (Figure 5). A detailed analysis of expression dynamics revealed that intronic transcripts more often postitively correlate with their paired protein coding transcripts when repression of the former is observed (Figures 10C, Figure 10D and Figure 11D). At the same time, positive correlation is predominantly observed for intronic lncRNAs with rapid (6 h post-induction), but not slow (120 h post-induction) activation.

The microarray array results gene pairs representative of all major GAs have been successfully validated by qRT-PCR. Our data indicate that intergenic lncRNA BC030713, encoded in the *HOXD *gene cluster between *HOXD3 *and *HOXD1*, was highly induced and positively correlated with *HOXD3 *expression. *HOXD *is one of the four *HOX *clusters of genes that are essential for embryonic development. These clusters are characterized by extensive network of lncRNA expression [28] with a concordant expression of lncRNAsand *HOX*genes. Interestingly, individual induction of several HOXD genes was sufficient to induce both growth arrest and neuronal differentiation, which is associated with downregulation of cell cycle-promoting genes and upregulation of neuronal differentiation genes [29]. The concordant expression of lncRNAs and HOX genes was also reported for the *HOXA *cluster during RA-induced differentiation of teratocarcinoma cell line [30] and for *HOXB *cluster during mouse embryonic stem cell differentiation [6]. Such co-regulated expressions may reflect shared regulatory elements of transcription or could be due to regulatory activity of lncRNA controlling the neighbouring genes. The transcriptional activity of *HOX *genes is highly dependent on chromatin modifications, such as active chromatin associated histone H3K4 trimethylation (H3K4me3) or repressing H3K27 trimethylation (H3K27me3) [31]. Indeed, the common regulatory theme of lncRNA-mediated control of *HOX *genes is recruitment of chromatin modifying complexes catalyzing these modifications, such as *cis*-activation of *HOXA *cluster by HOTTIP lncRNA-mediated recruitment of WDR5/MLL complex causing H3K4me3 modification [32], or trans-repression of *HOXD *genes by HOTAIR lncRNA recruiting PRC2 complex that imposes H3K27me3 mark [33].

Regarding the antisense GA, a comparable number of cases of positively and negatively correlating gene pairs were observed (Figure 5). Therefore, it is possible that both positive [34-36] and negative [37-39] regulatory mechanisms may act in such cases. Currently, the postive regulatory role of antisense transcripts is more favored in the scientific community. However, here we report a validated case of negative correlation of FW340058 lncRNA with the transcript of its tail-to-tail antisense protein-coding neighbor *PAICS *(Figure 12). PAICS catalyzes steps 6 and 7 of purine biosynthesis required for proliferation of cancer cells. PAICS is activated in many cancer types [40]. Arrest of neuroblastoma cells proliferation is preceded their differentiation.

In our study we noticed an unusual case of lncRNA AK096262 sharing a bidirectional promoter with *FKBP4 *protein-coding gene and negatively correlating with its transcription (Figure 12). The ligand of FKBP4, FK506 was shown to stimulate neuronal differentiation and induce the rapid regeneration of hippocampal neurons [41]. It is usually assumed that transcription of genes from bidirectional promoters correlates positively. Indeed, such antiregulated expression of genes, as negatively correlating *AK096262 -FKBP4 *gene pair is relatively rare in the human genome accounting for only 11% of cases [42]. This negative correlation may reflect the *cis*-regulatory effect of AK096262 on *FKBP4 *transcription, as has been reported for promoter associated lncRNAs targeting PRC2 complex that causes repressive histone trimethylation (H3K27me3) [43] for targeting inhibitors of activating histone deacetylases on the promoter of cyclin D1 gene [22]. An alternative model of inhibition of coding gene by non-coding transcription from bidirectional promoter is via the competition for the same pool of general transcription factors as was described for cryptic unstable transcripts (CUTs) regulating *TPI1 *gene expression in the yeast [44]. In this case the act of transcription is important *per se *rather than the functional activity of the transcription product.

Detailed GO analysis revealed that lncRNA genes have a tendency to co-localize with protein-coding genes having specific biological functions, such as embryonic development and cytoskeleton reorganization (see Additional file 1 Section 1.1). Moreover, these functional associations were specific for certain GA classes. By analyzing the dynamics of differentially expressed lncRNAs and their associated genes we found that these functions are realized by the cells via at least three gene expression waves. The first wave activated genes with housekeeping functions. The second wave was characterized by activation of homeotic transcription factors, PDGF pathway and actin cytoskeleton-related genes. Genes activated during the third wave encoded receptors, metabolism and signalling pathways specific to neural cells. The coordinated waves of gene expression have been reported for protein coding genes during neuronal differentiation of embryonic stem cells [45]. Our study is the first demonstration of such waves for lncRNA genes.

In sum, in the present paper we dissected a global picture of correlating expression between lncRNAs and their neighboring protein-coding genes. We provided a detailed analysis of genome architecture, which we regard as an active player in regulation of transcription. Working hypotheses regarding particular systemic mechanisms bridging together coding and noncoding parts of the genome to regulate cellular differentiation have been formulated. Future studies will be dedicated to their exprerimental testing.

## Conclusions

This is the first report detailing dynamical changes of multiple lncRNAs during RA-induced neuroblastoma differentiation. Integration of genomic and transcriptomic levels of information allowed us to demonstrate specific behavior of lncRNAs organized in different GAs. This study also provides a list of lncRNAs with possible roles in neuroblastoma.

## Methods

### Cell cultures

SH-SY5Y cell line was purchased from American Type Culture Collection (ATCC^®^ CRL-2266™). SH-SY5Y cells were cultured in Ham's F12 DMEM supplemented with 10% FBS in a humidified incubator at 37°C with 5% CO_2_. For cell differentiation experiment SH-SY5Y cells were plated onto laminin coated dishes and on the next day were induced to differentiate by addition of 10 μ*M *all-trans-retinoic acid (RA, Sigma).

### Custom microarray chip

Custom microarray (Agilent) was designed to measure expression of putative lncRNA transcripts annotated in H-Invitational database. 11,564 probes were designed to match a 60nt sequence at the 3'-end of the transcripts (see the full list in Additional file 11). The chip also included 42 quality control probes and 728 probes matching 446 protein-coding genes for cross-chip quality control. Total RNA from SH-SY5Y cell lysates was purified using RiboPure™ kit (Ambion/Life Technologies) according to the manufacturer's instructions. The quality of total RNA was assessed using an Agilent 2100 Bioanalyzer (Agilent Technologies) RNA samples were sent to DNA Chip Research Inc. (Yokohama, Japan) for hybridization.

### qRT-PCR validation

Total RNA was used as a template for reverse transcription using QuantiTect Reverse Transcription Kit (Qiagen) using random hexamer primers. The transcript levels were analyzed by qPCR run on Rotor-Gene Q machine using Rotor-Gene SYBR Green PCR Kit (Qiagen). The primers used throughout our study are listed in Additional file 12.

Computational analysis

### Probe filtering pipeline

From the list of probes, 12,173 non-redundant sequences have been selected. The sequences were scanned across human mRNA database (UCSC refMrna, 11 Oct 09) with NCBI BLAST allowing non-gapped alignments of 95% identity with no more than 1 mismatch. Thus, each of remaining 12,132 probes uniquely matched at least one mRNA sequence. 1,825 probes matching reverse-complementary sequences of mRNA transcripts were excluded from the analysis. Alignment of the remaining 10,307 probes with known RNA sequences (UCSC, all_mRNA and refSeqAli tables, excluding random chromosomes and haplotypes) selected 10,177 transcripts. 289 probes were removed as duplicated in their mRNA sequences. Probes matching protein-coding RNAs were excluded by the presence of the latter in refGene database (UCSC, Oct 09) or by sharing a strong sequence similarity with any known protein-coding RNAs (assessed with CRITICA software). The remaining 7,926 probes matching 9,267 non-coding transcripts were thus selected for further analysis. They were classified into 19 classes by the architecture of their genes in respect to their localization relative to their nearest protein-coding genes. A more general classification into 5 super-classes (intronic, promoter-associated, intergenic, bidirectional and antisense) was further derived by combining topologically similar architecture classes.

Architecture distribution was analyzed in a sequence of transcript filtration steps: 1) all lncRNAs present on the custom microarray, 2) lncRNAs differentially expressed (differentially expressed) in neuroblastoma differentiation course, 3) differentially expressed lncRNAs associated with any proteins, 4) differentially expressed lncRNAs associated with differentially expressed proteins, 5) differentially expressed lncRNAs positively or negatively correlating with their associated differentially expressed protein counterparts (listed in Additional files 13 and 14, respectively).

### Statistical analysis

By comparing the lncRNA distribution changes between each two of the sequential steps of filtration significantly over- and under-represented lncRNA GAs and dynamical modes were identified. Statistical significance of each individual class was assessed by hypergeometric test of the difference between its frequency among the lncRNAs left after a given filtration step versus the class frequency among the lncRNAs removed by the filtration. The frequencies were tabulated in two ways: 1) all members of a given class versus all non-members; 2) all members of a given class versus all members of another class. Thus two null-hypotheses of the frequency bias in lncRNAs was tested: 1) the frequency of a given class was not affected by the filtration procedure; 2) the frequency of a given class was not affected relative to the frequency of another particular class.

To remove the bias in the P-values resulting from multiple comparisons Bonferroni correction was applied.

### Analysis of gene expression

LncRNA expression in neuroblastoma cells was measured with the microarray at 4 time points (0, 6, 24 and 120 hours) following RA-induced differentiation. The experiment was repeated in two biological replicates. Fold change between the first and the last time point and Kendall's correlation coefficient were chosen as measures of differential expression and concordance. A gene was classified as differentially expressed if its expression in two biological replicates was concordant in any three of the four time points (Kendall's τ>0.6) and the fold change was not less than 1.5.

Classification of expression profiles into eight dynamical modes was performed in two ways. "Expression rate" modes the difference between the median sample expression value at a given time point was calculated relative to the previous time point, the magnitude of the rate was ignored, and the sign of the rate was considered. Thus each mode represented the sign of the rate of expression change between two sequential time points. Thus, for the four studied time points the eight modes were identified as all eight possible combinations of the rate signs between them (Additional file 2, panel A). The "magnitude modes" were defined as eight combinations of values 0 (low) and 1 (high) at the four time points of the experiment (Additional file 2, panel B). To classify a given gene expression timecourse by the modes Pearson's correlation coefficients between the expression values and each mode were calculated and the mode with the highest correlation was selected as the representative.

Initially, we attempted two normalization methods applicable for both Agilent and Affymetrix array types analyzed in our studies: i) RMA background correction [46] followed by quantile normalization with [47]. and ii) normalization by ACTB gene expression values. Due to the low number of replicates, use of both methods resulted in a notable artifacts. Therefore, to ensure the robustness of our results, we skipped the normalization procedure. Since the computational methods applied to analysis of gene expression are based on correlation measures, it does not result in false positive results.

### Gene ontology analysis

The functional classification of lncRNA genes was inferred from the available information on the GOs of the associated protein-coding genes. GO over-representation analysis was carried out using PANTHER database [48]. The P-values were calculated according to the binomial test integrated in the PANTHER online tool. Bonferroni's corrections for multiple testing was applied, followed by assessment of the GO significance at P-value cutoff level 0.05.

## List of abbreviations used

lncRNA: long noncoding RNA; GA: genomic architecture; FE: fold enrichment; RA: retinoic acid.

## Competing interests

The authors declare that they have no competing interests.

## Authors' contributions

IVK conceived the project. AB, PJ and YN carried out bioinformatics and computational work. AY and JZT obtained all of the experimental data. IVK contributed to data analysis and interpretation. AB and AY wrote the manuscript. All authors read and approved the final manuscript.

## Supplementary Material

Additional file 1 **Supplementary results**. Click here for file

Additional file 2 **List of rate (A) and magnitude (B) modes discriminated in the present study**. Click here for file

Additional file 3 **Distribution of lncRNA genomic architecture classes by rate dynamic modes**. Click here for file

Additional file 4 **Distribution of lncRNA genomic architecture classes by magnitude dynamic modes**. Click here for file

Additional file 5 **Distribution of lncRNA combined genomic architecture classes by rate dynamic modes for lncRNAs significantly correlating with associated differentially expressed proteins**. Note: absence of subfigures for certain lncRNA groups indicates insufficient statistics. Click here for file

Additional file 6 **Distribution of lncRNA combined genomic architecture classes by magnitude dynamic modes for lncRNAs significantly correlating with associated differentially expressed proteins**. Note: absence of subfigures for certain lncRNA groups indicates insufficient statistics. Click here for file

Additional file 7 **Distribution of lncRNA magnitude dynamic modes by combined genomic architecture classes for lncRNAs significantly correlating with associated differentially expressed proteins**. Note: absence of subfigures for certain lncRNA groups indicates insufficient statistics. Click here for file

Additional file 8 **Distribution of lncRNA rate dynamic modes by genomic architecture classes for lncRNAs significantly correlating with associated differentially expressed proteins**. Note: absence of subfigures for certain lncRNA groups indicates insufficient statistics. Click here for file

Additional file 9 **Distribution of lncRNA magnitude dynamic modes by genomic architecture classes for lncRNAs significantly correlating with associated differentially expressed proteins**. Note: absence of subfigures for certain lncRNA groups indicates insufficient statistics. Click here for file

Additional file 10 **Distribution of lncRNA rate dynamic modes by combined genomic architecture classes for lncRNAs significantly correlating with associated differentially expressed proteins**. Note: absence of subfigures for certain lncRNA groups indicates insufficient statistics. Click here for file

Additional file 11 **List of Agilent microarray probes**. Click here for file

Additional file 12 **List of primers used for validation studies with quantitative PCR**. Click here for file

Additional file 13 **List of lncRNAs in complex gene architecture positively correlating with their co-localized proteins**. Click here for file

Additional file 14 **List of lncRNAs in complex gene architecture negatively correlating with their co-localized proteins**. Click here for file

## Declarations

Publication of this article was funded by Bioinformatics Institute, Agency for Science, Technology and Research (A*STAR), Singapore.

This article has been published as part of *BMC Systems Biology *Volume 7 Supplement 2, 2013: Twelfth International Conference on Bioinformatics (InCoB2013): Bioinformatics. The full contents of the supplement are available online at http://www.biomedcentral.com/bmcsystbiol/supplements/7/S3.
