# Supplementary material for: Role of genomic architecture in the expression dynamics of long noncoding RNAs during differentiation of human neuroblastoma cells
Source: BMC Syst Biol. 2013 Oct 16;7(Suppl 3):S11. doi: 10.1186/1752-0509-7-S3-S11 (PMC3852107; doi:10.1186/1752-0509-7-S3-S11)
Supplement: Additional file 1 — Supplementary results. [file 1752-0509-7-S3-S11-S1.pdf]

1 Supplementary Results

1.1 Gene ontology analysis of lncRNA-protein coding gene pairs genomic architecture

Intronic architecture type defined the group of lnRNA genes strongly associated with neural differentiation processes, that were expected from the present experimental model. The enriched biological process GOs included visual perception (FE=4.1, $P = 2.57 \cdot 10{-}5$), synaptic transmission (FE=3.4, $P = 1.09 \cdot 10^{-5}$), neurological system process (FE=2.7, $P = 2.41 \cdot 10^{-10}$), nervous system development (FE=2.5, $P = 3.53 \cdot 10^{-4}$). Other GOs for this class of genes could rather be classified as generally found among cancer-related genes: angiogenesis (FE=4.1, $P = 2.0 \cdot 10^{-3}$), mesoderm development (FE=2.8, $P = 3.71 \cdot 10^{-8}$), cell motion (FE=2.5, $P = 1.45 \cdot 10^{-3}$). Finally, some GOs could be associated with both neural and cancer cells development: cell adhesion (FE=3.3, $P = 1.76 \cdot 10^{-11}$), cell-cell signalling (FE=2.4, $P = 3.67 \cdot 10^{-4}$), cell adhesion (FE=3.27, $P = 1.16 \cdot 10^{-11}$), cell communication (FE=2.2, $P = 9.82 \cdot 10^{-16}$). The functions of the proteins, which genes were associated with the intronic lncRNAs were enriched in receptors (FE=2.1, $P = 1.46 \cdot 10^{-4}$), structural molecules (FE=2.2, $P = 8.32 \cdot 10^{-4}$), lipid transporters (FE=5.8, $P = 3.49 \cdot 10^{-2}$), and calcium binding (FE=2.7, $P = 3.7 \cdot 10^{-2}$).

Among the bidirectional promoter-associated lncRNA genes only 5k-distant architecture type demonstrated a significant enrichment in specific biological functions. Anatomical structure morphogenesis was 4.4 times over-represented among these genes ($P = 2.85 \cdot 10^{-3}$). These genes included three transcription factors (LHX8 and LHX9 and FOXF2), protein modification enzymes (GAN, CSNK1G3, MAP3K9, MARK2 and MICAL3) and a protein kinase regulator (LPP). Most of the proteins encoded by these genes (LHX8, LHX9, GAN, MICAL3, and LPP) are localized in cytoskeleton. It was reflected in the observed enrichement of the given group of lncRNA genes in actin cytoskeleton (5.8 times, $P = 2.07 \cdot 10^{-2}$) and intracellular localization (3.7 times, $P = 2.19 \cdot 10^{-2}$).

Among the downstream-associated lncRNA genes located not further than 1kb away from the protein-coding genes, the significantly enriched biological processes were largely related with embryonic development: gut mesoderm development (FE=30, $P = 2.38 \cdot 10^{-2}$), segment specification (FE=22.2, $P = 4.34 \cdot 10^{-3}$), angiogenesis (FE=14.3, $P = 2.49 \cdot 10^{-2}$), muscle organ development (FE=10.2, $P = 1.60 \cdot 10^{-2}$), and embryonic development (FE=13.9, $P = 3.95 \cdot 10^{-3}$). In contrast, the only significant biologial process GO of the lncRNA genes associated in the intergenic architecture up to 10kb distances away from the protein-coding genes, was gamete generation (FE=4.7, $P = 4.41 \cdot 10^{-2}$).

Intergenic lncRNA genes were associated with MAPKKK cascade and spermiogenesis pathways were the

uniquely specific biological processes of the upstram 1k-distant genes (FE=4.7, $P = 3.59 \cdot 10^{-2}$) and the upstream 10k-distant genes (FE=5.4, $P = 7.75 \cdot 10{-}3$), respectively. Muscle organ development (downstream 5k-distant FE=4.7, $P = 9.97 \cdot 10^{-3}$) and regulation of transcription from RNA polymerase II promoters (FE=2.5, $P = 4.84 \cdot 10{-}2$) were the biological processes enriched among the intergenic 5k-distant genes.

Among the lncRNA genes associated with protein-coding genes in the exonic tail-to-tail antisense architecture, intracellular signalling cacade was the only significantly enriched biological process (FE=3.4, $P = 9.61 \cdot 10^{-2}$). In contrast, lncRNA genes embedded in the exons of protein-coding genes, due to four associated genes encoding myosins (MYH1, MYH2, MYH2, MYH8), were characterized with several significantly enriched GOs: sensory perception of sound (FE=57.1, $P = 7.80 \cdot 10{-}5$), cytokinesis (FE=36.4, $P = 4.6 \cdot 10^{-4}$), muscle organ development (FE=14.3, $P = 2.08 \cdot 10^{-2}$), muscle contraction (FE=12.9, $P = 2.94 \cdot 10^{-2}$), mitosis (FE=12.5, $P = 3.37 \cdot 10^{-2}$), cell motion (FE=9.6, $P = 1.49 \cdot 10{-}2$)., mesoderm development (the myosin genes, a transcription factor gene ERG, ubiquitin-protein ligase gene UBE3A, ubiquitin-protein ligase gene HECTD2; FE=9.5 $P = 2.78 \cdot 10^{-4}$), vesicle-mediated transport (FE=8.6, $P = 2.53 \cdot 10^{-2}$), intracellular signalling cascade (FE=7.31, $P = 9.80 \cdot 10^{-3}$).

1.2 Gene ontology analysis of dynamic patterns in lncRNA-protein coding gene pairs

Genes permanently activated by 24h were enriched in signal transduction ($P = 7.6 \cdot 10^{-11}$), cell communication ($P = 4.41 \cdot 10^{-10}$), developmental process ($P = 8.8 \cdot 10^{-7}$), mesoderm development ($P = 8.29 \cdot 10^{-5}$), neurological system process ($P = 3.66 \cdot 10^{-4}$), hemopoiesis ($P = 9.36 \cdot 10^{-3}$), and actin cytoskeleton ($P = 1.11 \cdot 10^{-4}$). Interestingly, no significant biological processes were revealed to be associated with genes activated by 6h.

In addition to the GOs specific to the gene pairs permanently activated at 24h, several GOs were uniquely enriched only among transiently activated pairs. They were: iIntracellular signalling cascade ($P = 5.26 \cdot 10^{-7}$), primary metabolic process ($P = 3.22 \cdot 10^{-4}$), cell adhesion ($P = 7.79 \cdot 10^{-3}$), muscle organ development ($P = 1.13 \cdot 10^{-2}$), ectoderm development ($P = 1.43 \cdot 10^{-2}$), nervous system development ($P = 1.51 \cdot 10^{-2}$), PDGF pathway ($P = 2.73 \cdot 10^{-2}$). Interestingly, the list of nerveous sytem development genes included nine transcription factors (HOXA11, HOXA13, JARID2, MAML3, NPAS3, ISX, ERG, MGAT5B, LHX8). PDGF pathway genes were represented by members of three levels of the signalling cascade: two enzymes of lipid metabolism (PIK3CB and PLCG2), two GTPase regulators (ARHGAP15 and SRGAP3), and two transcription factors (ERG, EHF), two protein kinases (PRKCA and MKNK1).

GO categories observed among the gene pairs activated by 120h were similar to the ones of the genes transiently ectivated at 24h. In addition, the former gene list contained the following significant GOs: cell-cell signalling ($P = 1.87 \cdot 10^{-5}$), synaptic transmission ($P = 3.36 \cdot 10^{-4}$), cation transport ($P = 7.78 \cdot 10^{-4}$), regulation of transcription from RNA polymerase II promoter ($P = 2.55 \cdot 10^{-3}$), cell motion ($P = 8.26 \cdot 10^{-3}$), primary metabolic process ($P = 1.67 \cdot 10^{-2}$), cell surface receptor linked signal transduction ($P = 3.08 \cdot 10^{-2}$), and sensory perception ($P = 3.98 \cdot 10^{-2}$). Genes belonging toSlit/Robo-mediated axon guidance pathway (RAC1, SLIT1, SLIT2, SLIT3 NTNG1, and NTN4) were significantly enriched ($P = 7.09 \cdot 10^{-3}$).

The functional specialization of the given pairs, belonging to the rate mode categories of transient (24h) and late (120h) activation, was also reflected in the corresponding magnitude modes. Protein coding genes associated with lncRNA genes transiently low expressed (6 to 24 hours post-induction) were enriched in cell communication ($P = 2.33 \cdot 10^{-2}$).

At the same time, analysis of the genes grouped by the rate modes revealed additional details of the lncRNA genes-associated protein coding gene expression. Gene pairs with high expression of lncRNA genes by 6h were enriched in metabolic process ($P = 7.28 \cdot 10^{-4}$) and catalytic activity ($P = 2.66 \cdot 10^{-4}$), nucleic acid binding ($P = 6.03 \cdot 10^{-3}$). Genes of the latter category included 19 transcription factors (RORB, FUS, IRX3, HMGB2, MLTT9, RUNX3, EPAS1, MYB, BCL11A, DLX1, NR6A1, TOX, CIITA, SOX6, PINX1, ZFPM1, DMRTC2, KLHL8 and EEF1B2), 19 DNA- and RNA-modifying enzymes (RAD1, DNASE1, POLD2, DCC1, DHX35, SRCAP, PINX1, ASH2L, LSM3, TERF1, PCNA, RCC1, PRPF19, XRCC5, LIG3, BICC1, HELLS, ADARB2, RAVER2), and 9 ribosomal proteins (MRPL3, RPL3, RPL5, RPL23, RPL39, MRPS18A, MRPS28, RPS19, NCL).

Genes with low expression established since 6h consistuted was the most abundant class of the lncRNA genes by dynamics, with 837 associated protein-coding genes mapped to known biologiical functions. were significantly enriched in the GOs similar to the ones of the gene pairs activated by 120h (see above). In addition, they were enriched in the following categories: skeletal system development ($P = 1.21 \cdot 10^{-3}$), endocytosis ($P = 3.66 \cdot 10^{-3}$), vitamin transport ($P = 6.57 \cdot 10^{-3}$), heart develpment ($P = 7.85 \cdot 10^{-3}$), visual perception ($P = 8.85 \cdot 10^{-3}$), cyclic nucleotide metabolic process ($P = 3.98 \cdot 10^{-2}$), sensory perception of sound ($P = 4.95 \cdot 10^{-2}$), and lipid metabolic process ($P = 4.99 \cdot 10^{-2}$). The list of 837 genes with available ontologies contained 132 transcription factors ($P = 7.30 \cdot 10^{-5}$), 25 of which belonged to homeobox family ($P = 7.93 \cdot 10^{-3}$), 44 actin family cytoskeletal proteins ($P = 4.45 \cdot 10^{-4}$), and 15 voltage-gated potassium channels ($P = 8.64 \cdot 10^{-3}$).

Genes with high expression estalished since 120h were enriched in vescle mediated transport ($P 2.03 \cdot 10^{-3}$),

EGF receptor (PHLDB2, YWHAZ, MAP3K3, RIT1, PRKCA, PIK3CD, AKT3, STAT4, and RHOQ genes; $P = 1.56 \cdot 10{-}2$) and Beta3 adrenergic receptor (GNB2, GNB5, GNG7, SNAP25, and STX3 genes; $P = 2.61 \cdot 10^{-2}$) signalling pathways, adrenaline biosynthesis (DDC, MAOB, SLC6A16, SLC6A17, SNAP25; $P = 4.63 \cdot 10^{-2}$), integrin signalling pathway ($P = 3.42 \cdot 10^{-2}$) and angiogenesis (RAP2A, RAP2C, ARHGAP26, COL5A1, ITGA6, ITGB3, MAP3K3, PARVA, PIK3CA, PIK3CD, RHOQ, TGFB1I1) $P = 4.68 \cdot 10^{-2}$).
