# Supplementary figures and images for: Role of genomic architecture in the expression dynamics of long noncoding RNAs during differentiation of human neuroblastoma cells
Source: BMC Syst Biol. 2013 Oct 16;7(Suppl 3):S11. doi: 10.1186/1752-0509-7-S3-S11 (PMC3852107; doi:10.1186/1752-0509-7-S3-S11)

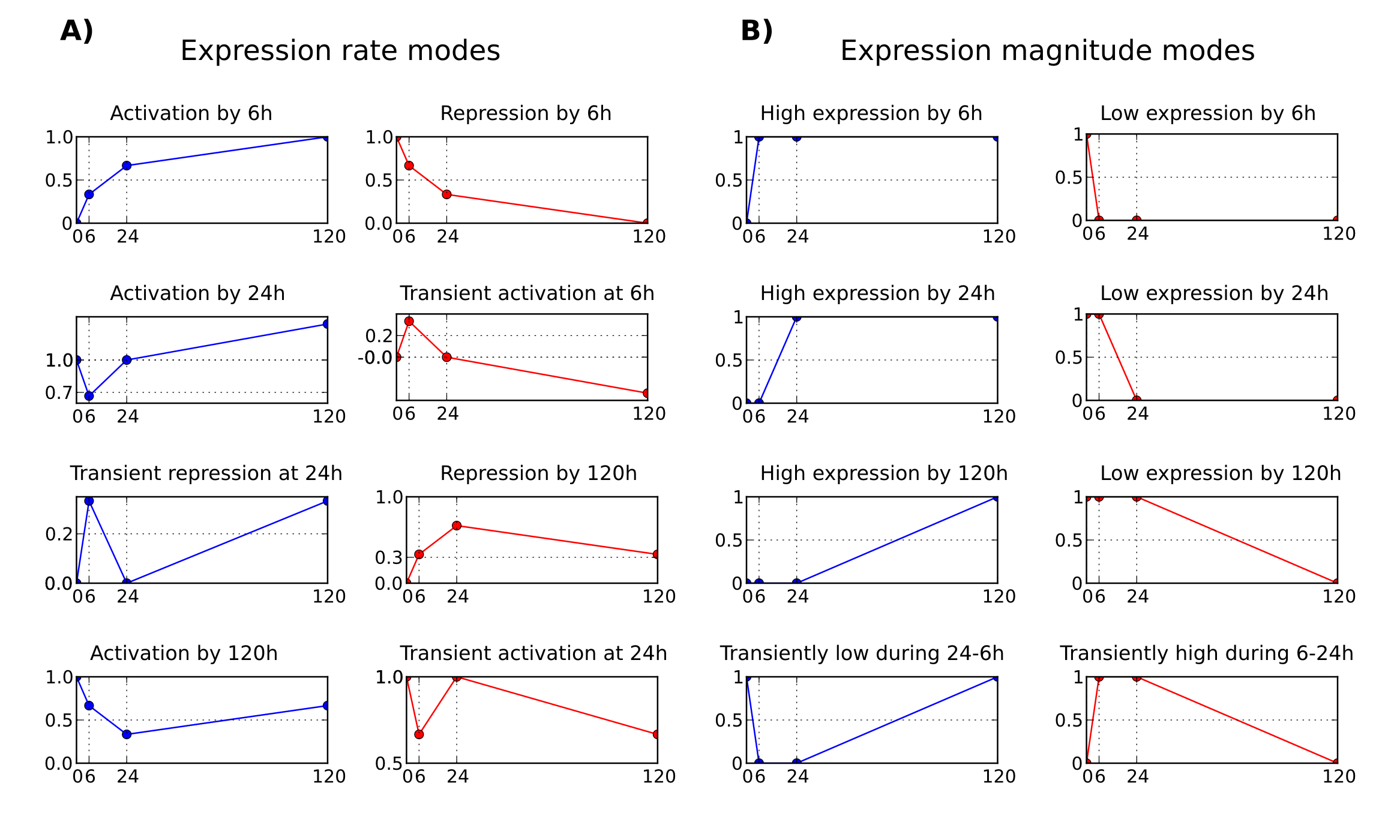

Supplement: Additional file 2 — List of rate (A) and magnitude (B) modes discriminated in the present study. [file 1752-0509-7-S3-S11-S2.png]

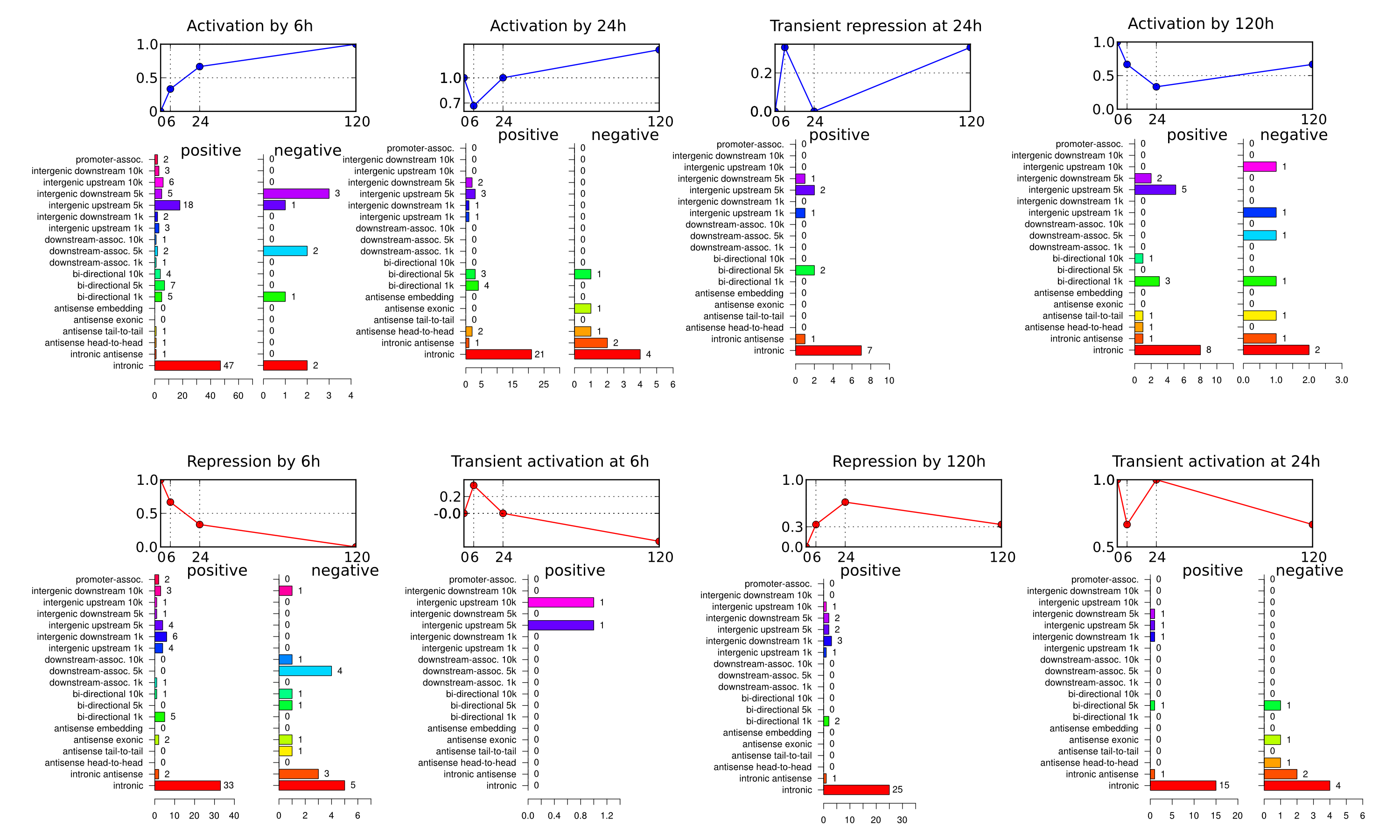

Supplement: Additional file 3 — Distribution of lncRNA genomic architecture classes by rate dynamic modes. [file 1752-0509-7-S3-S11-S3.png]

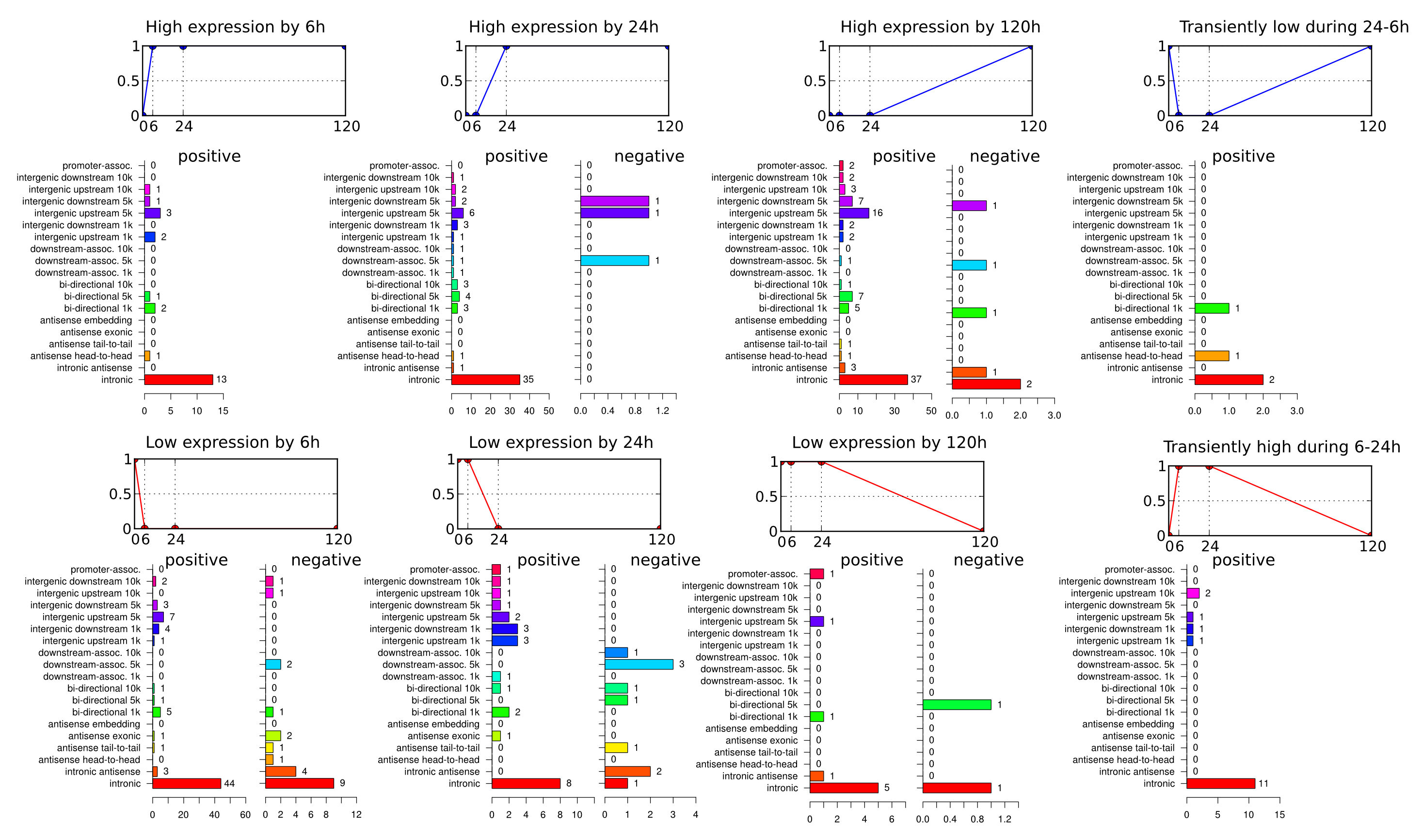

Supplement: Additional file 4 — Distribution of lncRNA genomic architecture classes by magnitude dynamic modes. [file 1752-0509-7-S3-S11-S4.png]

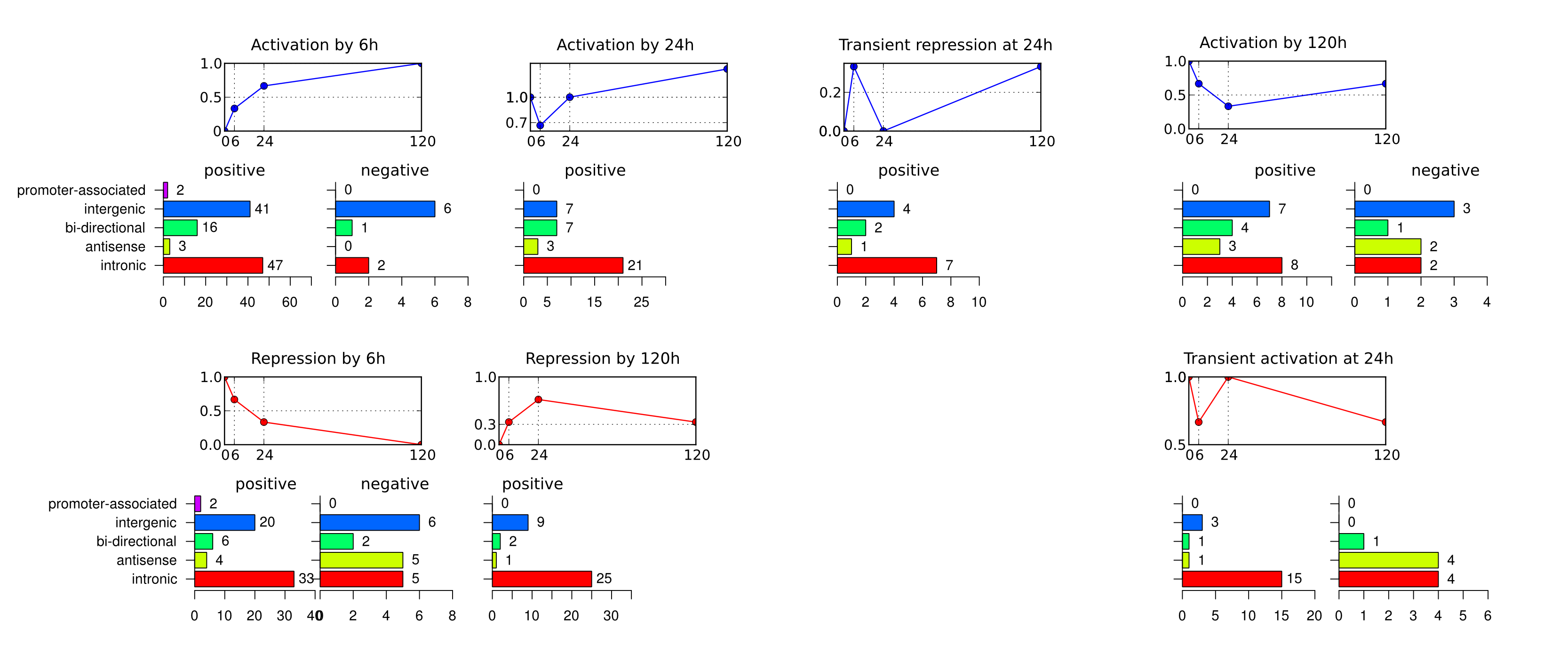

Supplement: Additional file 5 — Distribution of lncRNA combined genomic architecture classes by rate dynamic modes for lncRNAs significantly correlating with associated differentially expressed proteins. Note: absence of subfigures for certain lncRNA groups indicates insufficient statistics. [file 1752-0509-7-S3-S11-S5.png]

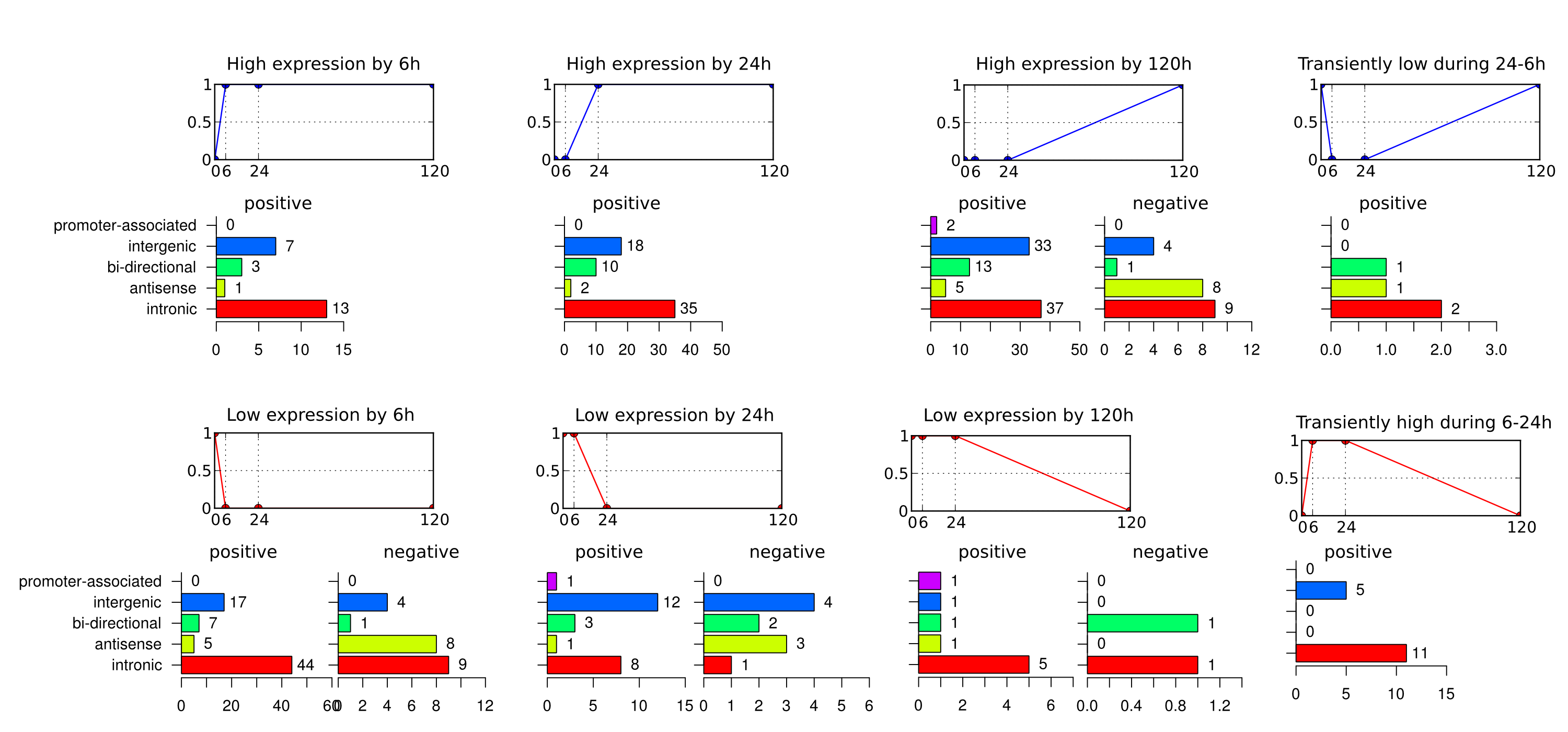

Supplement: Additional file 6 — Distribution of lncRNA combined genomic architecture classes by magnitude dynamic modes for lncRNAs significantly correlating with associated differentially expressed proteins. Note: absence of subfigures for certain lncRNA groups indicates insufficient statistics. [file 1752-0509-7-S3-S11-S6.png]

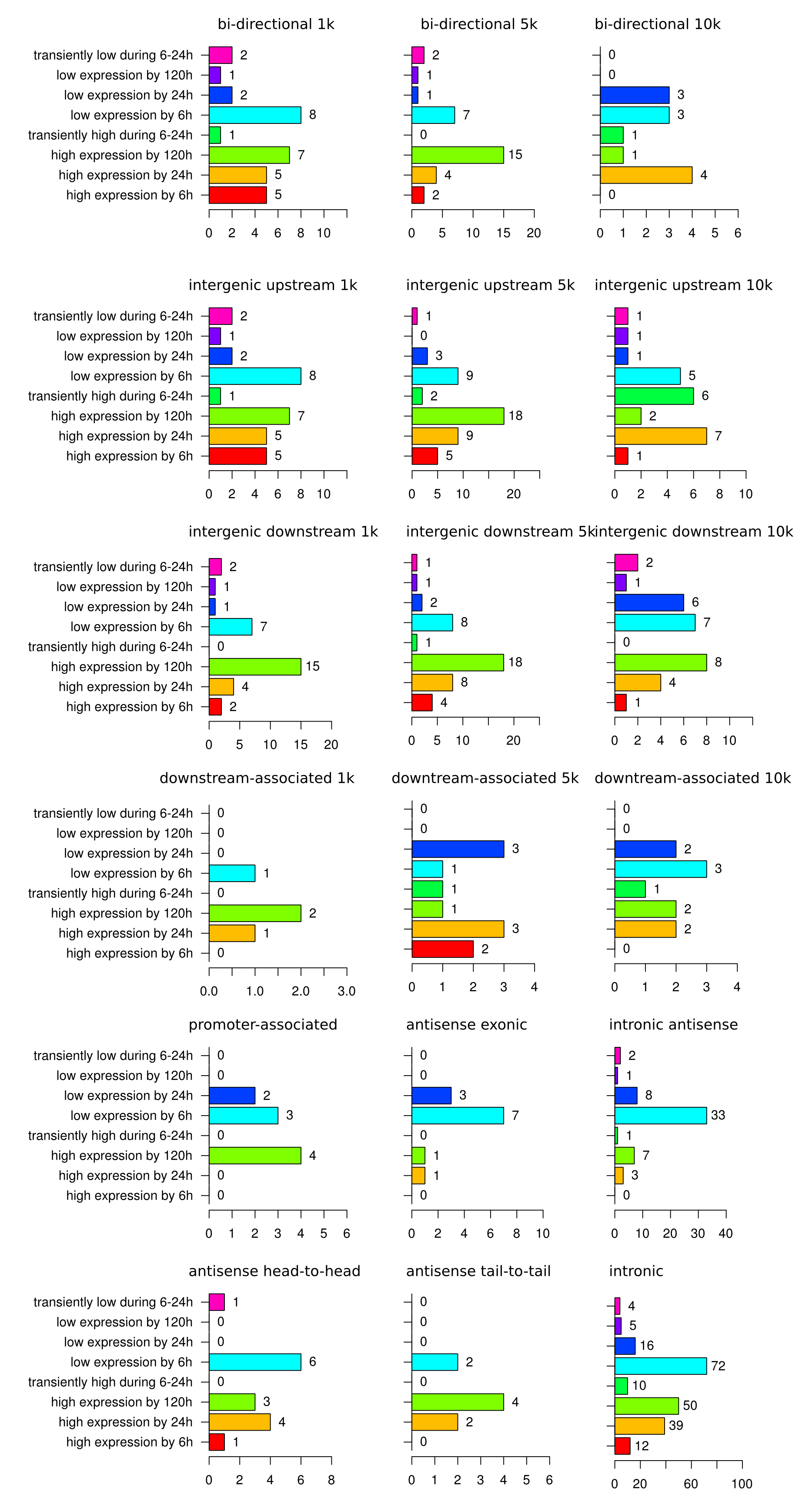

Supplement: Additional file 7 — Distribution of lncRNA magnitude dynamic modes by combined genomic architecture classes for lncRNAs significantly correlating with associated differentially expressed proteins. Note: absence of subfigures for certain lncRNA groups indicates insufficient statistics. [file 1752-0509-7-S3-S11-S7.png]

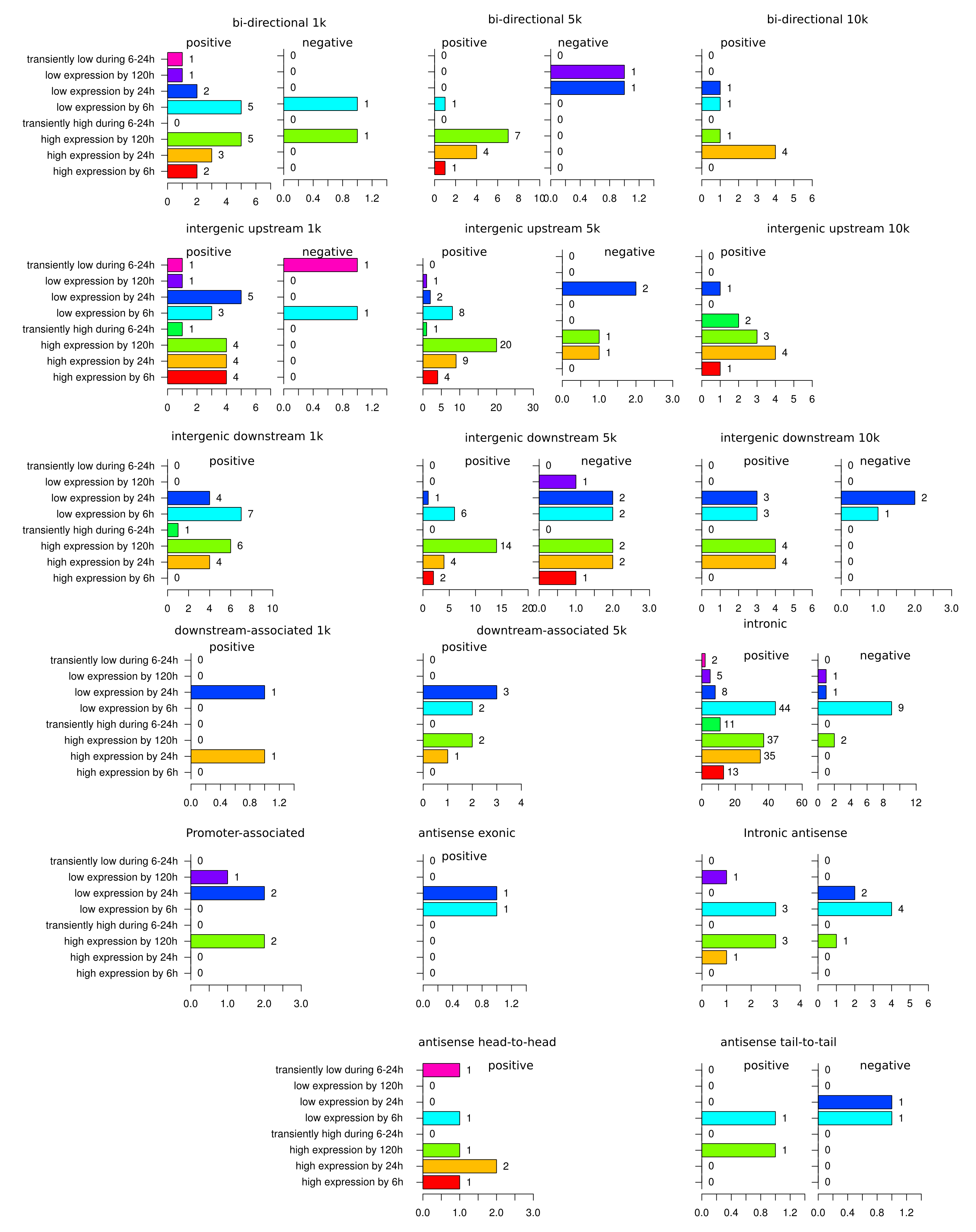

Supplement: Additional file 8 — Distribution of lncRNA rate dynamic modes by genomic architecture classes for lncRNAs significantly correlating with associated differentially expressed proteins. Note: absence of subfigures for certain lncRNA groups indicates insufficient statistics. [file 1752-0509-7-S3-S11-S8.png]

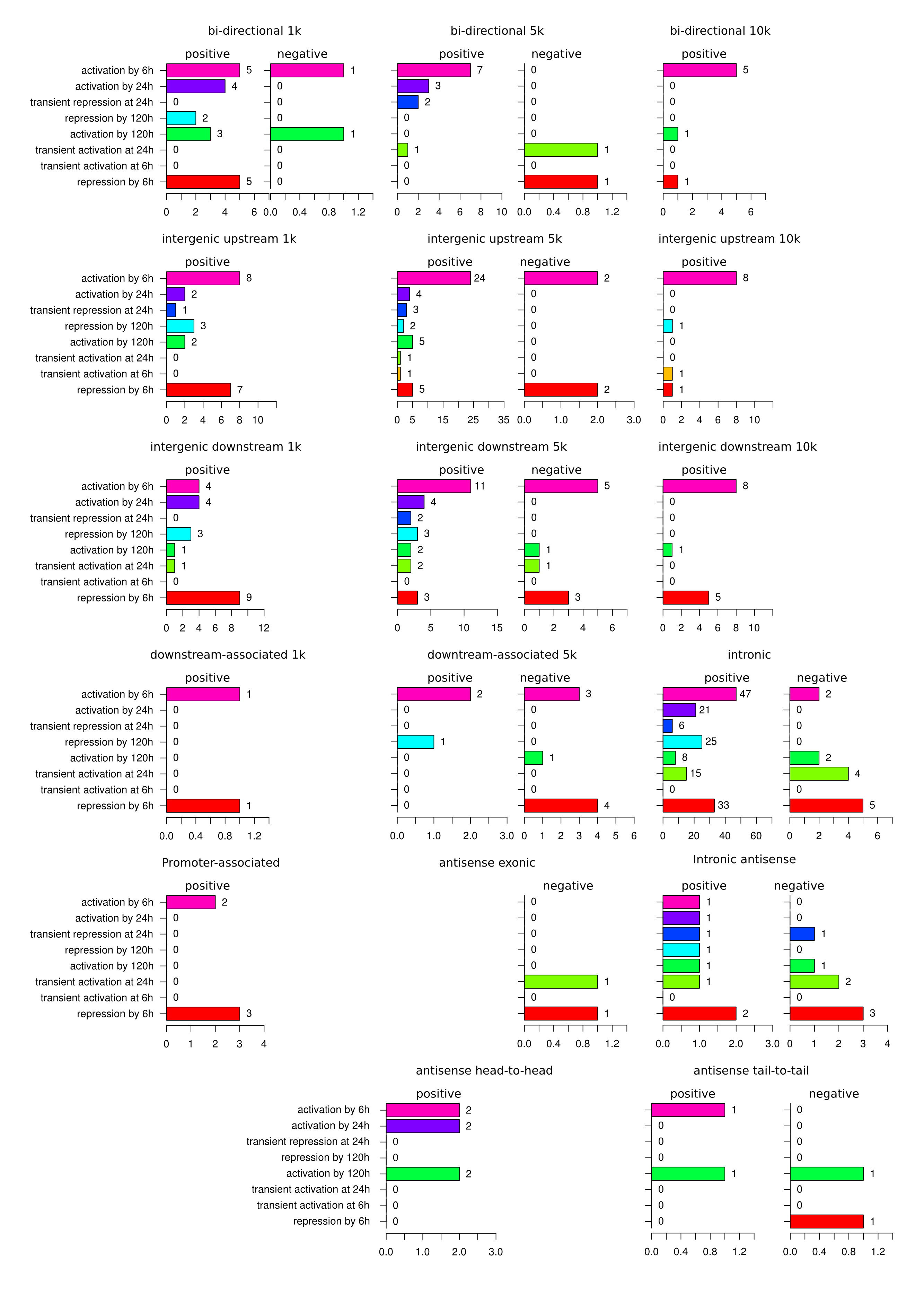

Supplement: Additional file 9 — Distribution of lncRNA magnitude dynamic modes by genomic architecture classes for lncRNAs significantly correlating with associated differentially expressed proteins. Note: absence of subfigures for certain lncRNA groups indicates insufficient statistics. [file 1752-0509-7-S3-S11-S9.png]

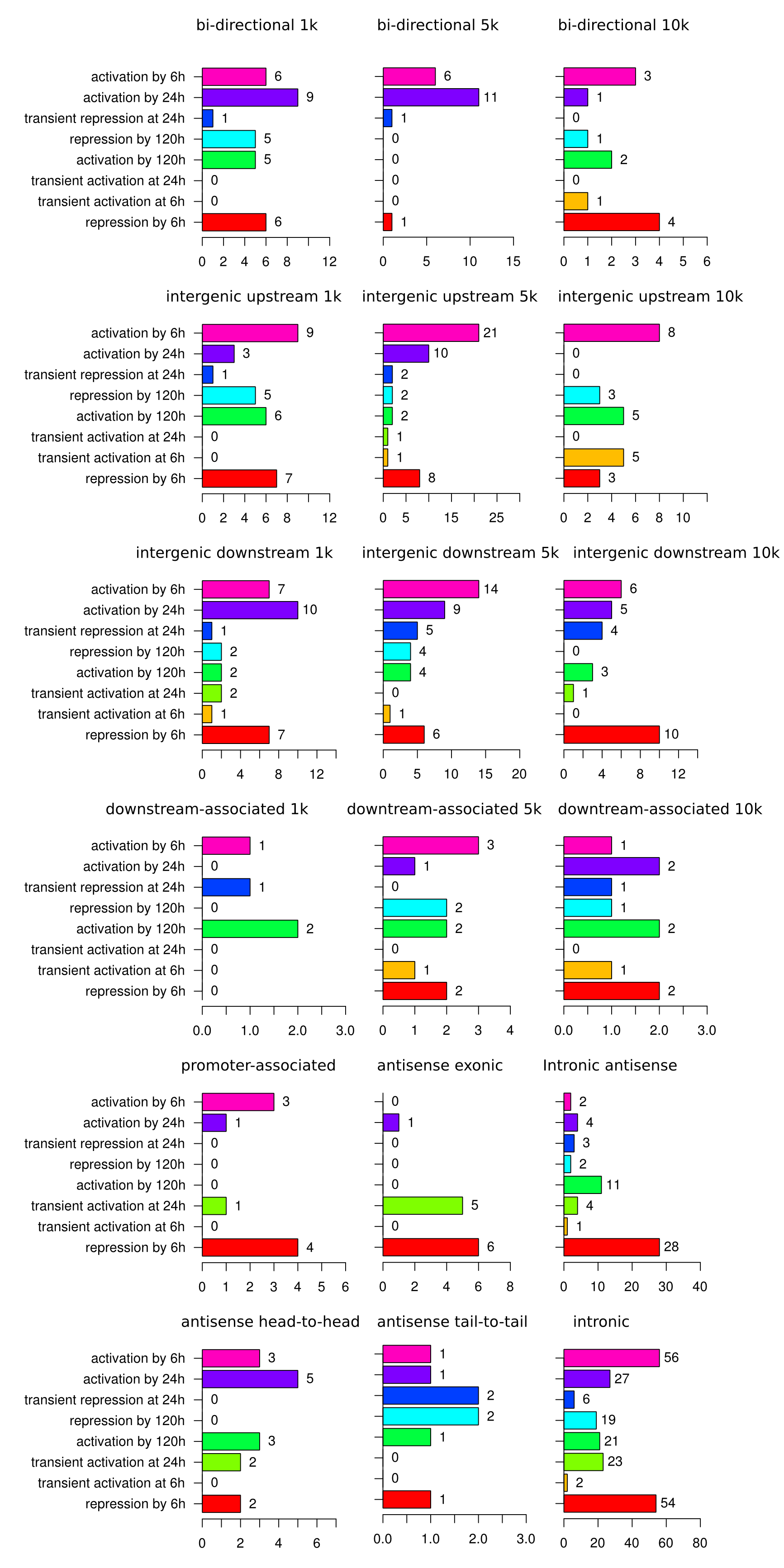

Supplement: Additional file 10 — Distribution of lncRNA rate dynamic modes by combined genomic architecture classes for lncRNAs significantly correlating with associated differentially expressed proteins. Note: absence of subfigures for certain lncRNA groups indicates insufficient statistics. [file 1752-0509-7-S3-S11-S10.png]
