# Supplementary material for: Role of genomic architecture in the expression dynamics of long noncoding RNAs during differentiation of human neuroblastoma cells
Source: BMC Syst Biol. 2013 Oct 16;7(Suppl 3):S11. doi: 10.1186/1752-0509-7-S3-S11 (PMC3852107; doi:10.1186/1752-0509-7-S3-S11)
Supplement: Additional file 12 — List of primers used for validation studies with quantitative PCR. [file 1752-0509-7-S3-S11-S12.pdf]

Transcript ID		Primer sequence
FKBP4	Forward	GAAGGCGTGCTGAAGGTCAT
	Reverse	TGCCATCTAATAGCCAGCCAG
AK096262	Forward	AAGAAAAAGCCCTTCGCTTC
	Reverse	AGCTGTCTCCAGCCACCTAA
PAICS	Forward	ATGGGTTTGCAGAAGAATAGCA
	Reverse	CACTGTGGGTCATTATTGGCAT
FW340058	Forward	ACTTGGGTTCTGGGGAAAGT
	Reverse	CCTTTCCTCACGTTTGCTTC
RORB	Forward	CGAGACCAGCGGCACTTATG
	Reverse	CCGGAATCGACGTTGTAATAACC
BC041938	Forward	TGTGGTTTCAGCAAGGCATA
	Reverse	TTTCTCCAAAAATACTCAGAAGGAA
BCL2	Forward	GGTGGGGTCATGTGTGTGG
	Reverse	CGGTTCAGGTACTCAGTCATCC
AK024118	Forward	TTGGCACAACAGGGTATTGA
	Reverse	TCCTTTGCCATAACAATGAGC
DBH	Forward	AGCCCAATATCCCCGAACC
	Reverse	GTAGCACCAGTACGTGGTCTC
BC067277	Forward	ACATCAGCCACTGCTGTGAT
	Reverse	TCTCCCTTGGATTTCAGGTG
HSPC111	Forward	AAAGAGCTTGTACGGAAGCCC
	Reverse	CCCGAGACAGAGTATTTCCTTTC
AF086071	Forward	GTCACCTTTGGAACCAGCAC
	Reverse	AGTTCACCTCCCACAACTGG

NOTCH2	Forward	GATCACCCGAATGGCTATGAAT
	Reverse	GGGGTCACAGTTGTCAATGTT
BC064338	Forward	CTTCACCACTTTGGCTTTGAG
	Reverse	CGTGTTGTTGCAAGTTGCTC
FAM69A	Forward	TGCTGTGAAAAGCTTCATTCC
	Reverse	GAACAGCAGCTTCAATGCAA
NR_002444	Forward	ACTAGCTCTGCGTGATGTGG
	Reverse	GGCTAACACATTTCCATCACC
HOXD3	Forward	CTAGCTCCTCAGCCACCATC
	Reverse	TCCTTTTCCAATTCCACCAG
BC030713	Forward	GGCTCTTCCCTAATGTGTGG
	Reverse	CAGGTCCAGCATGAAACAGA
GAPDH	Forward	CCATGAGAAGTATGACAACAGCC
	Reverse	GGGTGCTAAGCAGTTGGTG
